# Ultrasound-Assisted Synthesis and In Silico Modeling of Methanesulfonyl-Piperazine-Based Dithiocarbamates as Potential Anticancer, Thrombolytic, and Hemolytic Structural Motifs

**DOI:** 10.3390/molecules27154776

**Published:** 2022-07-26

**Authors:** Freeha Hafeez, Ameer Fawad Zahoor, Azhar Rasul, Asim Mansha, Razia Noreen, Zohaib Raza, Kulsoom Ghulam Ali, Ali Irfan, Gamal A. El-Hiti

**Affiliations:** 1 Department of Chemistry, Government College University Faisalabad, Faisalabad 38000, Pakistan; fariha5882@gmail.com (F.H.); mansha.asim@gmail.com (A.M.); kulsoom_gcu@yahoo.com (K.G.A.); raialiirfan@gmail.com (A.I.); 2 Department of Zoology, Government College University Faisalabad, Faisalabad 38000, Pakistan; drazharrasul@gmail.com; 3 Department of Biochemistry, Government College University Faisalabad, Faisalabad 38000, Pakistan; razianoreen@hotmail.com; 4 Department of Pharmacology, Government College University Faisalabad, Faisalabad 38000, Pakistan; zuhaib.raza@live.com; 5 Cornea Research Chair, Department of Optometry, College of Applied Medical Sciences, King Saud University, Riyadh 11433, Saudi Arabia

**Keywords:** anticancer, piperazine dithiocarbamates, hemolysis, in silico modeling, lung carcinoma, ultrasound

## Abstract

Piperazine-based dithiocarbamates serve as important scaffolds for numerous pharmacologically active drugs. The current study investigates the design and synthesis of a series of dithiocarbamates with a piperazine unit as well as their biological activities. Under ultrasound conditions, the corresponding piperazine-1-carbodithioates **5a**–**5j** were synthesized from monosubstituted piperazine **2** and *N*-phenylacetamides **4a**–**4j** in the presence of sodium acetate and carbon disulfide in methanol. The structures of the newly synthesized piperazines were confirmed, and their anti-lung carcinoma effects were evaluated. A cytotoxic assay was performed to assess the hemolytic and thrombolytic potential of the synthesized piperazines **5a**–**5j**. The types of substituents on the aryl ring were found to affect the anticancer activity of piperazines **5a**–**5j**. Piperazines containing 2-chlorophenyl (**5b**; cell viability = 25.11 ± 2.49) and 2,4-dimethylphenyl (**5i**; cell viability = 25.31 ± 3.62) moieties demonstrated the most potent antiproliferative activity. On the other hand, piperazines containing 3,4-dichlorophenyl (**5d**; 0.1%) and 3,4-dimethylphenyl (**5j**; 0.1%) rings demonstrated the least cytotoxicity. The piperazine with the 2,5-dimethoxyphenyl moiety (**5h**; 60.2%) showed the best thrombolytic effect. To determine the mode of binding, in silico modeling of the most potent piperazine (i.e., **5b**) was performed, and the results were in accordance with those of antiproliferation. It exhibits a similar binding affinity to PQ10 and an efficient conformational alignment with the lipophilic site of PDE10A conserved for PQ10A.

## 1. Introduction

Lung carcinoma is one of the most well-known causes of cancer death in many countries [1,2]. The annual death rate from lung carcinoma is double that of all other tumors combined [3,4]. The incomplete development of early detection methods is largely responsible for lung carcinoma’s uncontrolled spread [5]. Smoking is the leading cause of lung carcinoma worldwide [6]. Early diagnosis of other tumors, such as colon carcinoma, cervical carcinoma, and esophageal carcinoma, increases the survival rate [7]. It is vital to develop novel drug candidates for this type of carcinoma [8].

**Figure 1:**
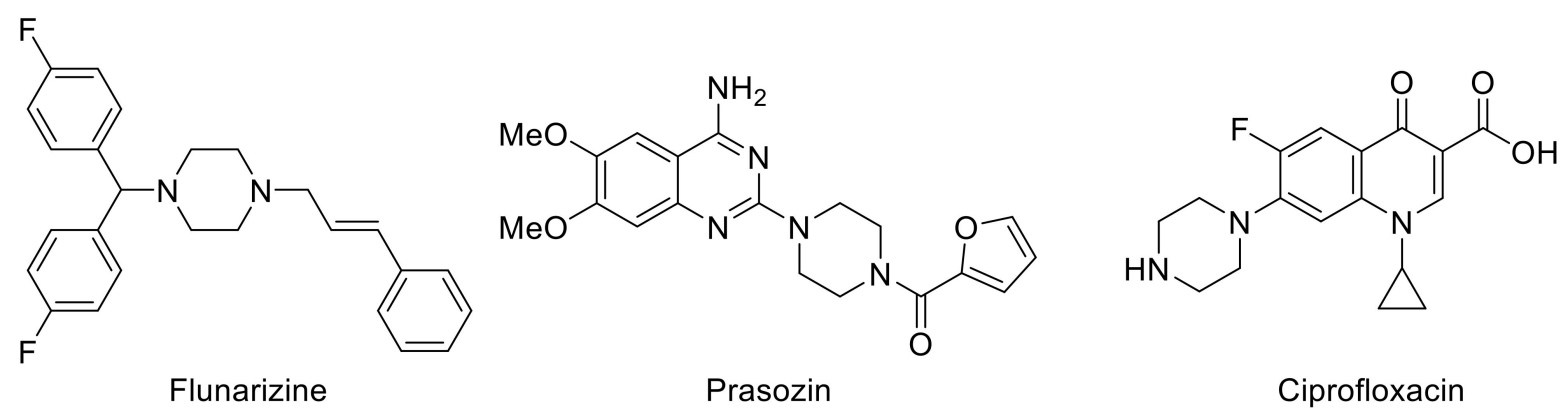
Structures of some common piperazine-based drugs.

Structurally modified nitrogen heterocycles have a broad spectrum of biological activities [9]. For example, heterocycles containing the piperazine moiety have a significant role in drug discovery programs [10]. Due to their vast array of pharmaceutical applications, *N*-substituted piperazines have garnered attention in academia and the pharmaceutical industry. They exhibit antimicrobial [11], antimycobacterial [12], antidepressant [13], and anticancer [14,15] properties. Previous reports revealed that *N*-substituted piperazines are a crucial structural component of the anticancer effect [16,17]. The presence of polar nitrogen in the piperazine framework is an essential component of the skeletons of numerous biologically active drugs [18]. Figure 1 shows the structures of some common piperazine-based drugs.

**Figure 2:**
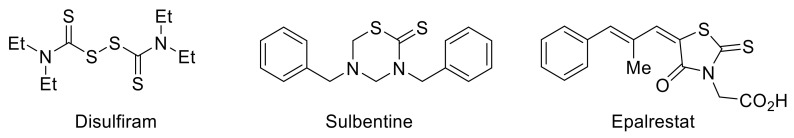
Structures of dithiocarbamate based drugs.

Dithiocarbamates are important intermediates in the synthesis of many pharmacologically active molecules [19,20]. They are active ingredients in numerous drugs (Figure 2), as well as naturally occurring and synthesized anticancer agents [21,22]. Including a dithiocarbamate moiety in the skeleton of different organic compounds elevates the biological profile of the synthesized derivatives [23].

The incorporation of two or more biologically active molecules to create new hybrid structures with potential medicinal applications is a useful approach [24]. This strategy is adopted in an effort to enhance the biological profile of newly synthesized hybrids compared to individual molecules included in their skeletons [25,26]. In continuation of our research on hybrid molecules with cytotoxic potential, we now report the design, synthesis, and antiproliferative effect of piperazine-based acetamides against lung carcinoma (A-549).

## 2. Results and Discussion

### 2.1. Chemistry

**Scheme 1:**
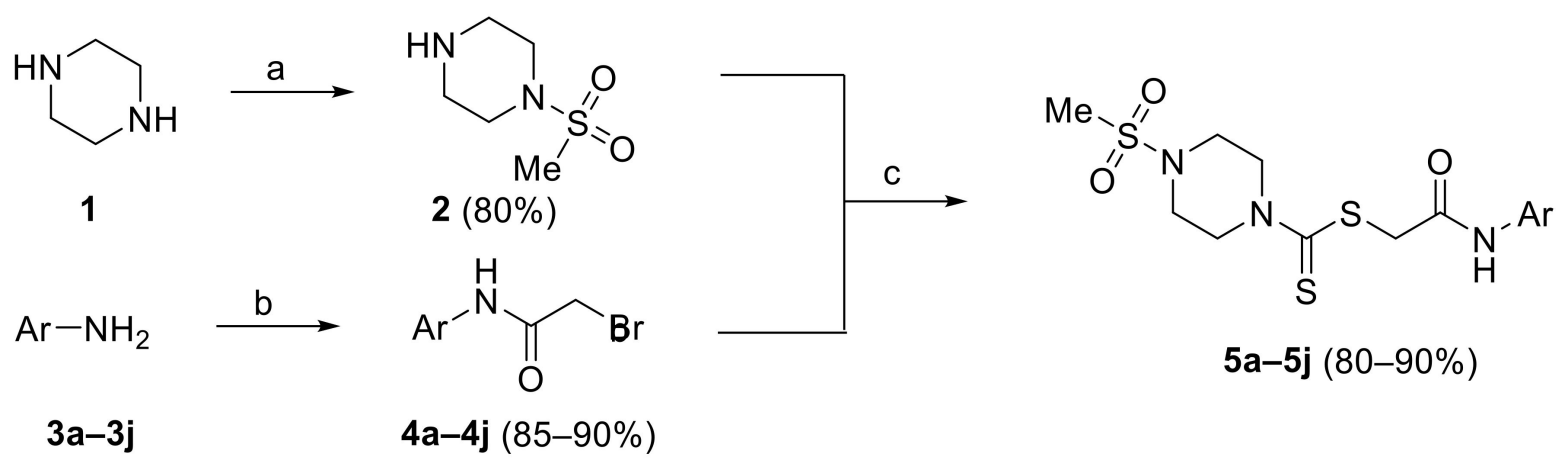
Synthesis of piperazine-1-carbodithioates **5a**–**5j**. Reagents and conditions: (**a**): methanesulfonyl chloride, DCM, 0 °C, (**b**): BrCH_2_COBr, pyridine, DCM, and (**c**): AcONa, CS_2_, MeOH, 70 °C, 30 min.

Several piperazine-dithiocarbamate hybrids **5a**–**5j** were synthesized, as shown in Scheme 1. The treatment of excess piperazine (**1**) with methane sulfonyl chloride at 0 °C in dichloromethane (DCM) produced monosubstituted piperazine **2** in an 80% yield [27]. Treating aryl amines **3a**–**3j** in a basic medium with bromoacetyl bromide afforded the corresponding *N*-phenyl acetamides **4a**–**4j** [28,29]. Reactions of **2** and **4a**–**4j** in the presence of sodium acetate (AcONa) and carbon disulfide (CS_2_) in methanol (MeOH) for 12–18 h at room temperature produced **5a**–**5j** in 55–65% yields. Piperazine-1-carbodithioates **5a**–**5j** were obtained in excellent yields (80–90%) when reactions of **2** and **4a**–**4j** were carried out under ultrasonic conditions at 70 °C for 30 min.

The structures of piperazine-1-carbodithioates **5a**–**5j** were established using a variety of spectroscopic techniques, and their purity was confirmed by elemental analyses (see the experimental section for details). The NMR spectra of **5a**–**5j** are shown in Figures S1–S20. The HRMS confirms the chemical formula of each derivative’s molecular ion peak. The FTIR spectra showed the presence of absorption bands corresponding to the NH (3230–3330 cm^−1^), C=O (1670–1660 cm^−1^), C=C (1530–1520 cm^−1^), and C=S (1225–1220 cm^−1^) groups. The NMR spectra of **5a**–**5j** contained all of the expected protons and carbons. At high fields, the ^1^HNMR spectra of **5a**–**5j** revealed the presence of methyl protons of the sulfonyl group (2.85–2.79 ppm) and methylene protons (4.36–4.20 ppm), as well as piperazine and aryl protons. At very low fields, the ^13^CNMR spectra revealed the presence of C=S (197.4–196.6 ppm) and C=O (167.0–163.9 ppm) carbons.

### 2.2. Antiproliferative Potential

The anticancer potential of newly synthesized piperazine-1-carbodithioates **5a**–**5j** against the human lung cancer cell line (A-549) was investigated using the MTT assay [30]. The cell viability of **5a**–**5j** varied depending on the aryl ring substituents (Table 1).

The SAR analysis of **5a**–**5j** was carried out to determine the role of various substituents on the aryl ring. Compounds **5b** and **5i** produced the best results, as shown in Table 1. The most potent derivative was compound **5b**, which contains a chlorine substituent at the *ortho*-position of the aryl ring and has a cell viability of 25.11 ± 2.49. The cell viability of compound **5i**, which contains two methyl groups at the *ortho* and *para* positions of the aryl ring, was comparable to that of compound **5b**, at 25.31 ± 3.62. Compounds **5d**, **5f**, and **5h** exhibited a significant effect, but it was lower than **5b** and **5i**. Compound **5e**, with a fluorine atom at the *ortho*-position of the aryl ring, showed the lowest potency and highest cell viability (68.94 ± 6.64), followed by **5c** (60.29 ± 5.96), which has a chlorine substituent at the *para*-position of the aryl ring.

**Figure 3:**
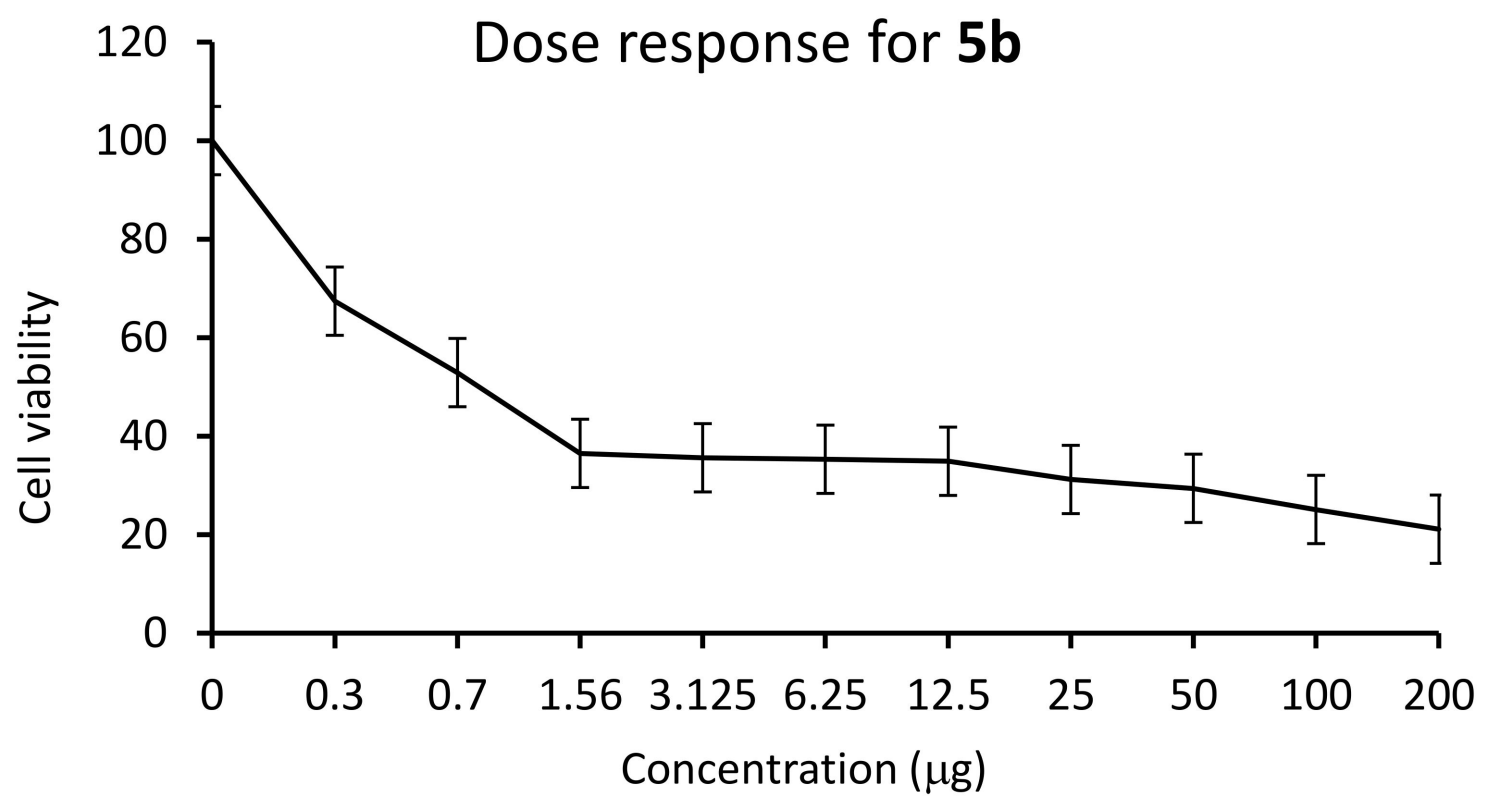
Dose-response of compound **5b**.

**Figure 4:**
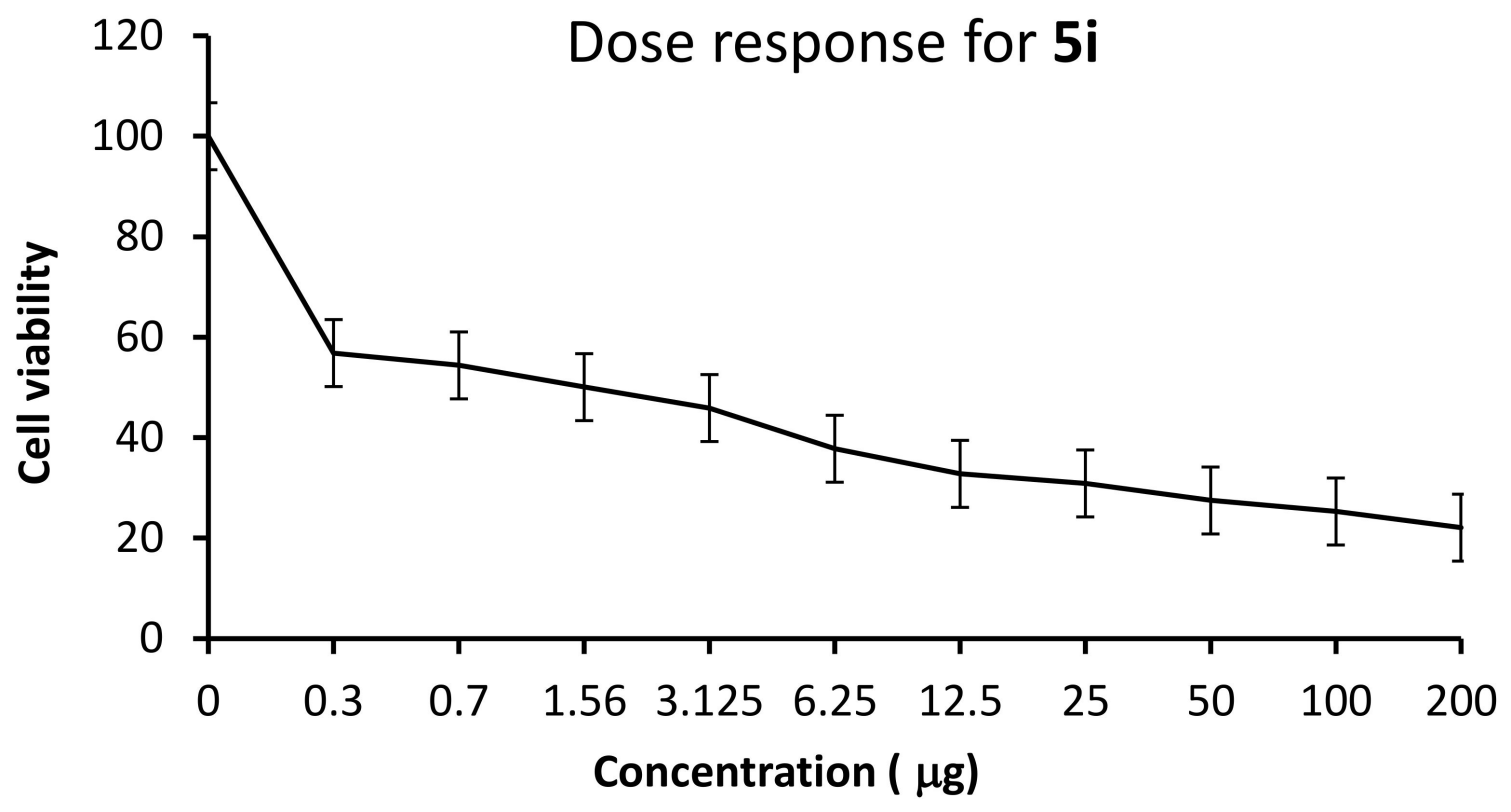
Dose-response of compound **5i**.

The cell viability of **5b** and **5i** was investigated further using various concentrations (0.3–200 µg) to test the dose-response relationship. Figure 3 and Figure 4 show that **5b** and **5i** produced the best results at a dose of 200 µg.

### 2.3. Hemolytic Potential

The cytotoxic activity of **5a**–**5j** was investigated using a reported methodology [31]. The age hemolysis (%) shown in Table 1 indicated that **5a**–**5j** had a low cytotoxic profile. The age hemolysis (%) for compound **5c** was 10%, demonstrating its binding affinity for hemoglobin, followed by **5h** (7%). Compounds **5a** (3.1%), **5b** (1.27%), **5f** (2.8%), **5g** (1.3%), and **5i** (2.8%) exhibited low toxicity. Compounds **5e** (0.3%), **5d**, and **5j** (0.1%) had the lowest cytotoxicity when compared to ABTS (95.9%).

### 2.4. Thrombolytic Potential

Cerebral venous sinus thrombosis (CVST) is a common central nervous system disorder caused by thrombophilia syndrome. Due to its anticoagulant profile, heparin is regarded as an effective treatment for CVST. Many thrombolytic agents are now used to treat thrombophilia syndrome. A reported methodology was used to evaluate compounds **5a**–**5j** for thrombolysis [31]. Compound **5h**, which contains a methoxy group at the *ortho*-position of the aryl ring, showed the highest potential (60.2%). Compounds **5a** (58.9%), **5i** (57.4%), and **5j** (58.7%) showed moderate thrombolytic potential. Compounds **5c** (47.8%), **5f** (50.3%), and **5g** (50.9%) had a mild effect compared to the positive control (i.e., ABTS; 80%).

### 2.5. Structure-Activity Relationship (SAR) Study

The effect of aryl ring substituents in compounds **5a**–**5j** on anticancer activity against A-549 was investigated. Compound **5b,** with Cl at the *ortho*-position of the aryl ring, exhibited the lowest cell viability and thus proved to be a potent cytotoxic agent. The replacement of Cl in **5b** (cell viability = 25.11 ± 2.49) with hydrogen (i.e., **5a**; cell viability = 61.35 ± 2.29) or fluorine (i.e., **5e**; cell viability = 68.94 ± 6.64) resulted in a significant decrease in cytotoxic potential. However, the substitution of Cl in **5b** with a fluorine atom at the *para*-position of the aryl ring (i.e., **5f**; cell viability = 41.01 ± 3.73) or a chlorine atom at the *para*-position (i.e., **5c**; cell viability = 60.29 ± 5.96) led to a sharp decrease in anticancer activity. In contrast, the insertion of two Cl at the *meta* and *para*-positions of the aryl ring (i.e., **5d**; cell viability = 38.08 ± 2.85) enhances anticancer activity. Inserting a methoxy group at the *para*-position of the aryl ring (i.e., **5g**; cell viability = 43.36 ± 4.42), on the other hand, led to a significant change in anticancer activity. The two methoxy substituents at the *ortho* and *meta*-positions in compound **5h** (cell viability = 40.25 ± 3.34) are responsible for the good antiproliferative potential when compared to **5e,** which has a methoxy group at the *ortho*-position of the aryl ring. However, the replacement of the Cl in **5b** with two methoxy groups at the *ortho* and *para*-positions of the aryl ring (i.e., **5i**; cell viability = 25.31 ± 3.62) resulted in better activity when compared to the case where two methyl groups were present at the *meta* and *para*-positions of the aryl ring (i.e., **5j**; cell viability = 47.94 ± 1.57).

The trend for SAR studies related to hemolysis (Table 1) suggested that the presence of the chlorine atom at the *para*-position of the aryl ring (i.e., **5c**) showed the highest cytotoxic potential (10%) among the others. Compounds **5d** and **5j** exhibited the lowest cytotoxic potential (0.1%) for hemolysis, implying that the Cl and OMe substituents at the *ortho* and *para*-positions of the aryl ring have no affinity for hemoglobin. When compared to **5d** and **5j**, the two methoxy substituents at the *ortho* and *meta*-positions of the aryl ring (i.e., **5h**; 7.1%) increased the cytotoxic activity. However, the unsubstituted phenyl ring (i.e., **5a**) has 30 times more cytotoxic activity (3.1 %).

**Figure 5:**
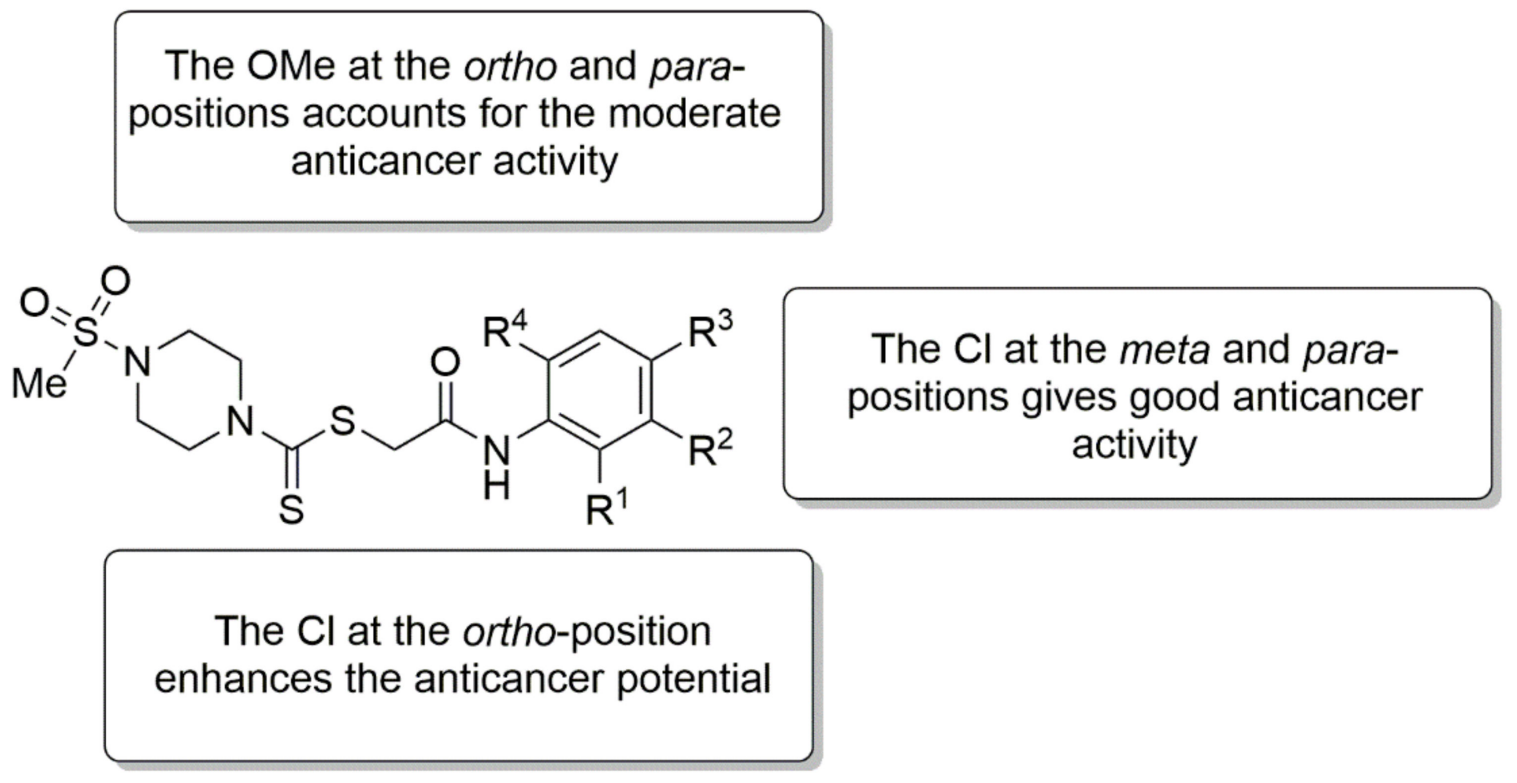
SAR summary.

Based on the data from the SAR study of piperazine-1-carbodithioates **5a**–**5j**, it is concluded that the presence of the Cl motif at the *ortho*-position of the aryl ring played a significant role in the antiproliferative activity. The presence of chlorine, dimethyl, and methoxy substituents improves anticancer activity (Figure 5).

### 2.6. In Silico Modeling

**Figure 6:**
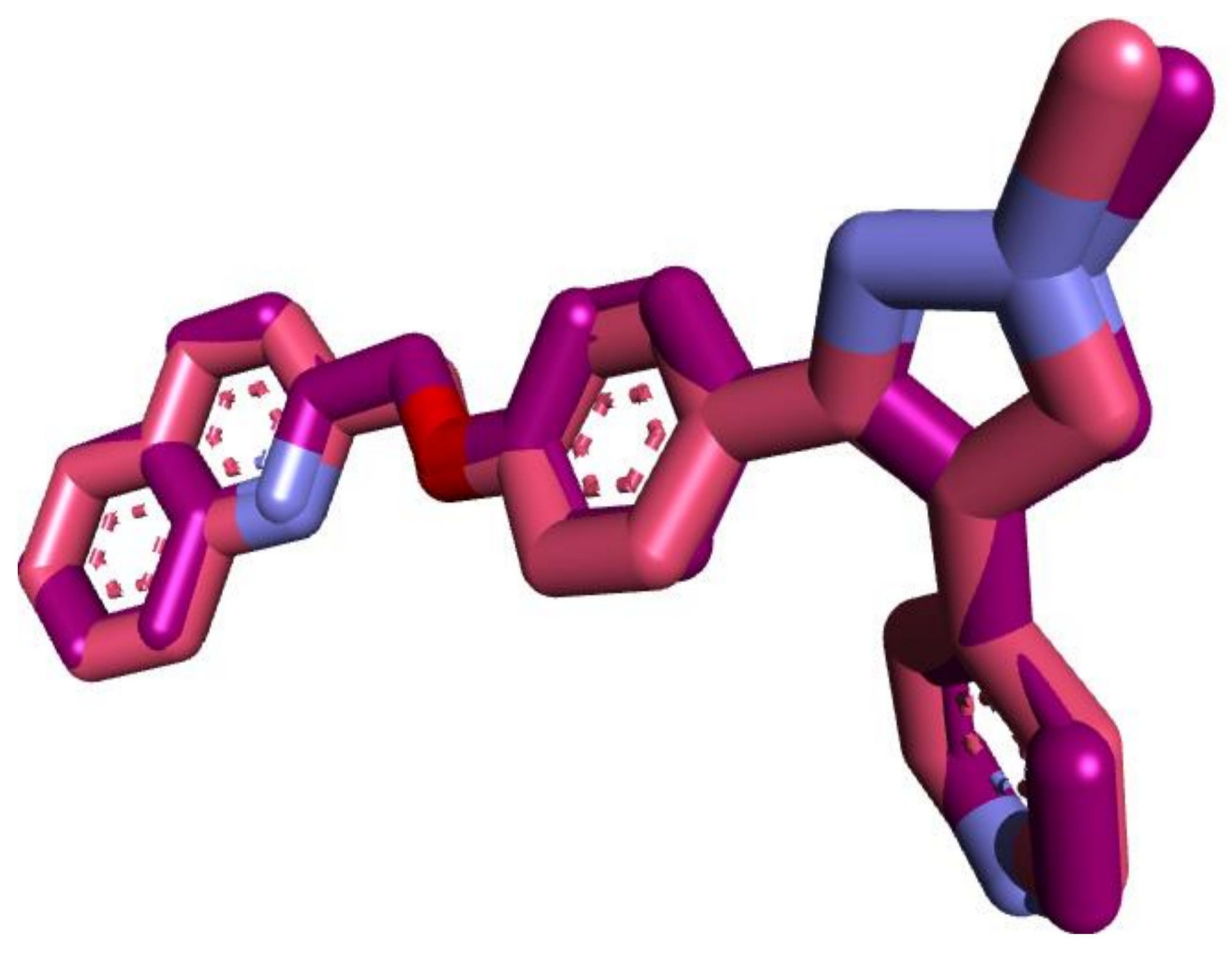
Validation of docking protocol by cognate redocking of the co-crystallized ligand of 3HR1; co-crystallized conformation (pink) compared to the re-docked conformation (purple) of native ligand in three-dimensional space.

The mode of action of compound **5b** was investigated further in silico. The SwissTargetPrediction identified phosphodiesterase 10A (PDE10A) as a potential target for **5b** with the highest (~1600) active similarity and 0.105 probability. The IFD method was validated by cognate re-docking, which resulted in a conformation with 0.2519 Å RMSD compared to the co-crystallized native conformation (Figure 6).

The IFD was carried out to investigate the binding of **5b** with its potential therapeutic target and simulate its binding conformation into the catalytic site. The binding free energy analysis revealed that compound **5b** binds at the catalytic site of PDE10A with ΔG of −8.30 kcal/mol which is comparable to −8.97 kcal/mol for the PQ-10 as a standard for PDE10A (Table 2).

**Figure 7:**
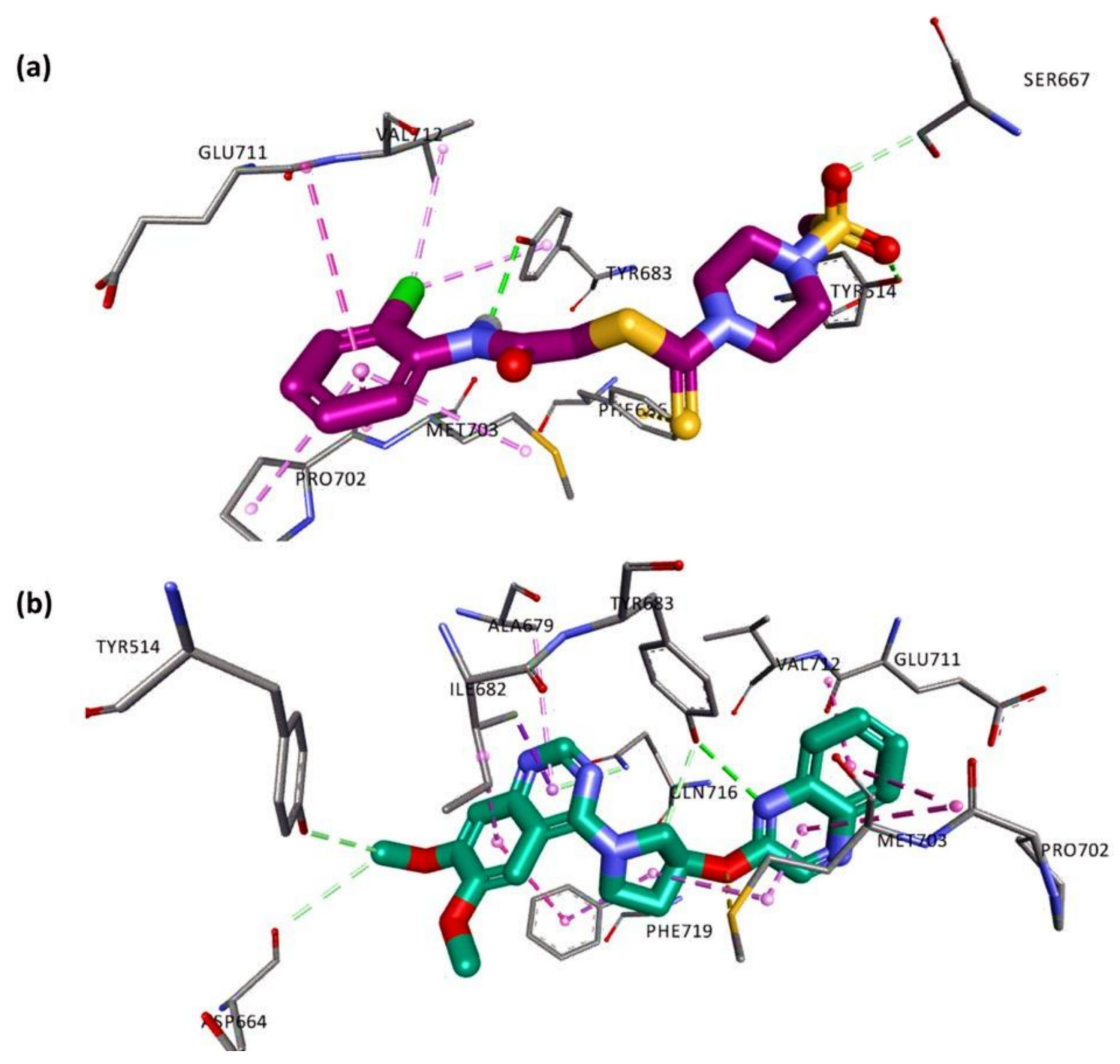
Conformational analysis of ligands docked within the binding pocket of PDE10A. The simulated best binding mode with the lowest ΔG of (**a**): PQ-10 and (**b**) compound **5b** within the 3D space of the PDE10A active site.

The conformational analysis of compound **5b** highlighted its efficiency in attaining a binding pose that established the diverse interactions with the vital residues within the catalytic pocket of PDE10A (Figure 7). Interestingly, **5b** shares similar interaction patterns to PQ-10. It is efficiently penetrated and anchored into the lipophilic pocket of PDE10A within the catalytic site which is inaccessible in all other isoforms of PDE. Therefore, the comparable binding behavior and affinity may suggest that **5b** exhibits a similar activity compared to PQ-10.

**Figure 8:**
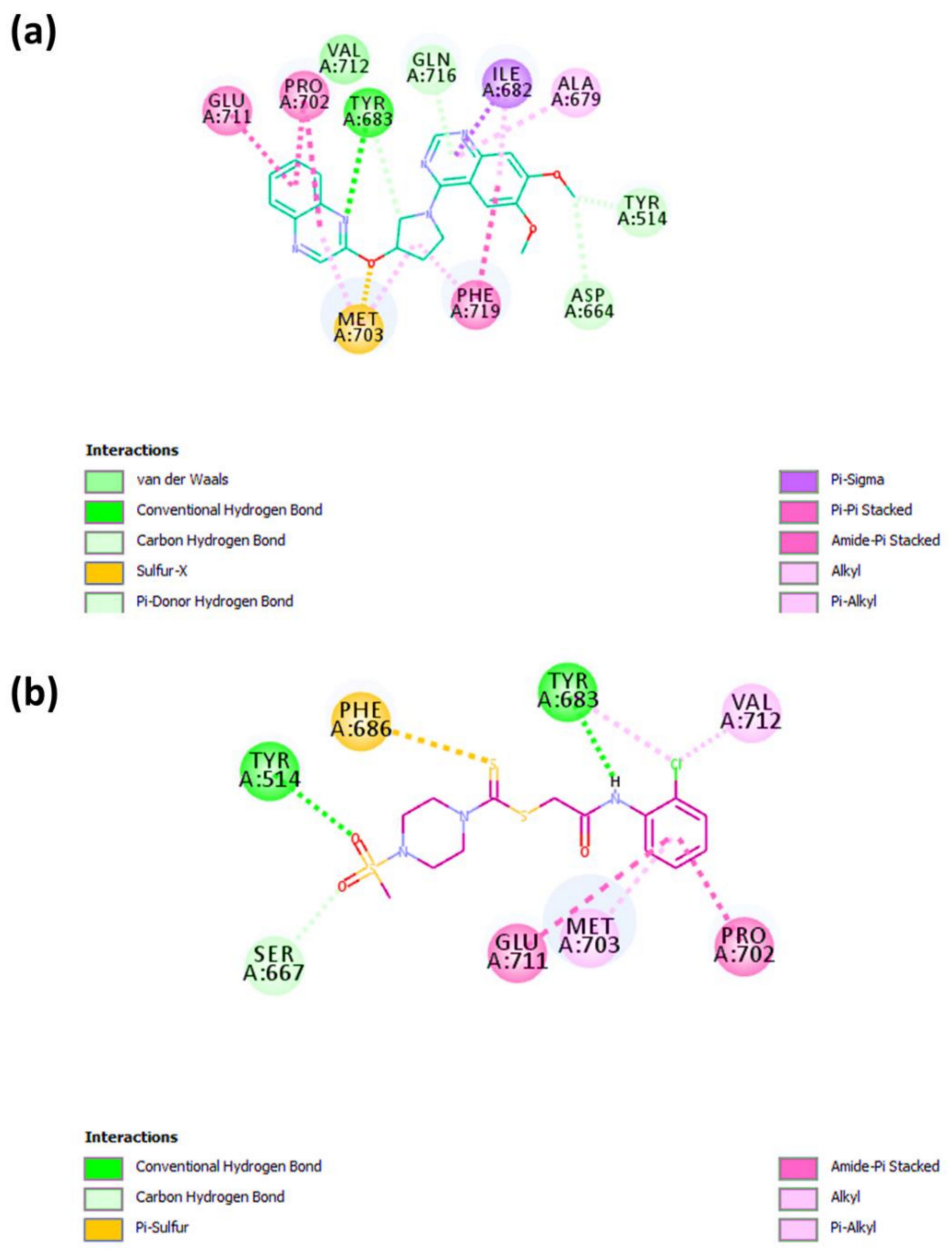
The Interaction behavior of ligands within the active site of PDE10A; (**a**) a 2D representation of PQ10 and (**b**) compound **5b** interacting with the PDE10A catalytic residues colored by type of interactions.

The conformational analysis was extended to delineate the complexation or anchoring of ligands within the binding pocket of PDE10A. The PQ-10 established H-bonds with TYR683, GLN716, VAL712, ASP664, and TYR514 to strengthen the stabilization of its confirmation within the catalytic site of PDE10A (Figure 8). This core stabilization was further supported by its extensive hydrophobic interactions with GLU711, PRO702, ILE682, PHE719, and ALA679 within the lipophobic cavity of PDE10A. Notably, **5b** interacts with conserved PQ10 residues. Compound **5b**’s conformation is stabilized by H-bonds with TYR683, TYR514, and SER667. The hydrophobic interactions between GLU711, MET703, PRO703, and VAL712 further stabilized this conformation. Furthermore, **5b** does not interact with GLN726 conserved in other PDE isoforms, resulting in greater selectivity for PDE10A.

Compound **5b** exhibits a binding affinity comparable to that of PQ10 and an efficient conformational alignment with the lipophilic site of PDE10A conserved for PQ10A. In addition, **5b** establishes interactions with the conserved PQ10 residues, but with a distinct bonding pattern, implying potential differences in activity and selectivity. This study provides valuable insight into the potential therapeutic activity of **5b** against PDE10A.

## 3. Materials and Methods

### 3.1. General

Chemicals, solvents, and reagents were purchased from Merck (Gillingham, UK) and used without further purification. Melting points were recorded on a Gallenkamp instrument (Fisons; Uckfield, UK). The ^1^H (400 MHz) and ^13^C NMR (100 MHz) spectra were recorded in deuterated chloroform (CDCl_3_) using a Bruker DPX spectrophotometer (Bruker; Zürich, Switzerland). The chemical shifts were recorded in ppm related to either tetramethylsilane or CDCl_3_. Thin-layer chromatography (DCM/MeOH) was utilized in conjunction with a Spectroline E-Series UV lamp to monitor the progression of chemical reactions (Merck; Gillingham, UK). Compound **2** [27] and *N*-phenyl acetamides **4a**–**4j** [28,29] were produced based on reported procedures.

### 3.2. General Procedures for the Synthesis of Piperazine-1-carbodithioates ***5a***–***5j***

#### 3.2.1. Conventional Method

A solution of appropriate *N*-phenylacetamides **4a**–**4j** (0.6 mmol) in dry MeOH (0.3 mL) was added to a stirred mixture of **2** (0.10 g, 0.6 mmol), anhydrous AcONa (0.36 g, 0.60 mmol), and CS_2_ (0.39 g, 0.6 mmol) in dry MeOH (0.8 mL). The mixture was stirred at 20 °C for 12–18 h according to the TLC. The produced solid was collected, washed with H_2_O, dried, and recrystallized from EtOH to give pure **5a**–**5j** as white solids in moderate to good yields (55–65%).

#### 3.2.2. Ultrasound-Assisted Method

The procedure was identical to that described in Section 3.2.1. with the exception that the mixture was sonicated at 70 °C for 30 min. Following crystallization, pure **5a**–**5j** was obtained in excellent yields (80–90%) as white solids.

#### 3.2.3. 2-Oxo-2-(phenylamino)ethyl 4-(methylsulfonyl)piperazine-1-carbodithioate (**5a**)


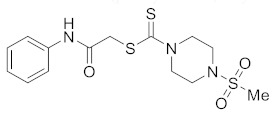


Yield 90%, m.p. 188–189 °C. IR: ν (cm^−1^): 3230 (NH), 1664 (C=O), 1524 (C=C), 1473 (CH_2_), 1222 (C=S). ^1^H NMR (*δ*): 8.79 (s, 1H, NH), 7.48 (d, 2H, *J* = 8.0 Hz, Ar), 7.29 (t, 2H, *J* = 8.0 Hz, Ar), 7.08 (t, 1H, *J* = 8.0 Hz, Ar), 4.48 (br s, 2H, piperazinyl), 4.22 (s, 2H, SCH_2_), 4.10 (br s, 2H, piperazinyl), 3.36 (t, 4H, *J* = 4.0 Hz, piperazinyl), 2.79 (s, 3H, Me). ^13^C NMR (*δ*): 197.4 (C=S), 166.8 (C=O), 137.8, 129.6, 124.7, 120.1, 45.7 (NCH_2_), 45.4 (NCH_2_), 40.7 (SMe), 35.5 (SCH_2_). HRMS: *m*/*z* [M]^+^ calculated for C_14_H_19_N_3_O_3_S_3_: 373.0589; found: 373.0582. Analysis calculated for C_14_H_19_N_3_O_3_S_3_: C, 45.02; H, 5.13; N, 11.25; found: C, 45.07; H, 5.17; N, 11.28%.

#### 3.2.4. 2-((2-Chlorophenyl)amino)-2-oxoethyl 4-(methylsulfonyl)piperazine-1-carbodithioate (**5b**)


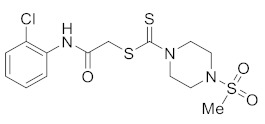


Yield 82%, m.p. 180–181 °C. IR: ν (cm^−1^): 3330 (NH), 1662 (C=O), 1524 (C=C), 1470 (CH_2_), 1220 (C=S). ^1^H NMR (*δ*): 8.80 (s, 1H, NH), 8.36 (d, 1H, *J* = 8.0 Hz, Ar), 7.37 (d, 1H, *J* = 8.0 Hz, Ar), 7.28 (t, 1H, *J* = 8.0 Hz, Ar), 7.06 (t, 1H, *J* = 8.0 Hz, Ar), 4.55 (br s, 2H, piperazinyl), 4.32 (s, 2H, SCH_2_), 4.24 (br s, 2H, piperazinyl), 3.14 (t, 4H, *J* = 4.0 Hz, piperazinyl), 2.84 (s, 3H, SMe). ^13^C NMR (*δ*): 197.4 (C=S), 166.7 (C=O), 134.9, 129.5, 128.3, 125.2, 123.5, 121.8, 50.0 (NCH_2_), 45.3 (NCH_2_), 40.9 (SMe), 35.3 (SCH_2_). HRMS: *m*/*z* [M]^+^ calculated for C_14_H_18_ClN_3_O_3_S_3_: 407.0199; found: 407.0209. Analysis calculated for C_14_H_18_ClN_3_O_3_S_3_: C, 41.22; H, 4.45; N, 10.30; found: C, 41.26; H, 4.48; N, 10.35%.

#### 3.2.5. 2-((4-Chlorophenyl)amino)-2-oxoethyl 4-(methylsulfonyl)piperazine-1-carbodithioate (**5c**)


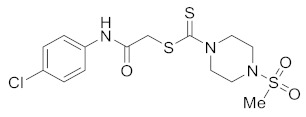


Yield 87%, m.p. 200–201 °C. IR: ν (cm^−1^): 3230 (NH), 1662 (C=O), 1525 (C=C), 1470 (CH_2_), 1225 (C=S). ^1^HNMR (*δ*): 8.89 (s, 1H, NH), 7.43 (d, 2H, *J* = 8.0 Hz, Ar), 7.26 (d, 2H, *J* = 8.0 Hz, Ar), 4.49 (br s, 2H, piperazinyl), 4.21 (s, 2H, SCH_2_), 4.97 (br s, 2H, piperazinyl), 3.38 (t, 4H, *J* = 4.0 Hz, piperazinyl), 2.84 (s, 3H, SMe). ^13^C NMR (*δ*): 197.4 (C=S), 167.0 (C=O), 136.1, 129.6, 129.3, 121.4, 45.6 (NCH_2_), 45.3 (NCH_2_), 4073 (SMe), 35.4 (SCH_2_). HRMS: *m*/*z* [M]^+^ calculated for C_14_H_18_ClN_3_O_3_S_3_: 407.0199; found: 407.0209. Analysis calculated for C_14_H_18_ClN_3_O_3_S_3_: C, 41.22; H, 4.45; N, 10.30; found: C, 41.25; H, 4.46; N, 10.33%.

#### 3.2.6. 2-((3,4-Dichlorophenyl)amino)-2-oxoethyl 4-(methylsulfonyl)piperazine-1-carbodithioate (**5d**)


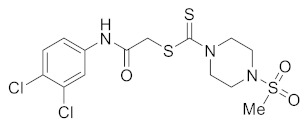


Yield 86%, m.p. 162–163 °C. IR: ν (cm^−1^): 3235 (NH), 1667 (C=O), 1528 (C=C), 1470 (CH_2_), 1222 (C=S). ^1^H NMR (*δ*): 8.89 (s, 1H, NH), 7.69 (s, 1H, Ar), 7.33 (d, 1H, *J* = 8.0 Hz, Ar), 7.24 (d, 1H, *J* = 8.0 Hz, Ar), 4.48 (br s, 2H, piperazinyl), 4.20 (s, 2H, SCH_2_), 4.12 (br s, 2H, piperazinyl), 3.37 (t, 4H, *J* = 4.0 Hz, piperazinyl), 2.81 (s, 3H, SMe). ^13^C NMR (*δ*): 197.4 (C=S), 166.7 (C=O), 137.4, 132.9, 130.6, 127.7, 121.5, 119.2, 45.6 (NCH_2_), 45.4 (NCH_2_), 40.4 (SMe), 35.6 (SCH_2_). HRMS: *m*/*z* [M]^+^ calculated for C_14_H_17_Cl_2_N_3_O_3_S_3_: 440.9809; found: 440.9812. Analysis calculated for C_14_H_17_Cl_2_N_3_O_3_S_3_: C, 38.01; H, 3.87; N, 9.50; found: C, 38.05; H, 3.87; N, 9.54%.

#### 3.2.7. 2-((2-Fluorophenyl)amino)-2-oxoethyl 4-(methylsulfonyl)piperazine-1-carbodithioate (**5e**)


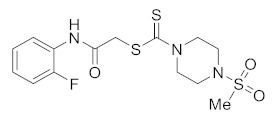


Yield 84%, m.p. 182–183 °C. IR: ν (cm^−1^): 3250 (NH), 1670 (C=O), 1530 (C=C), 1475 (CH_2_), 1225 (C=S). ^1^H NMR (*δ*): 8.80 (s, 1H, NH), 8.27 (d, 1H, *J* = 8.0 Hz, Ar), 7.08 (m, 2H, Ar), 7.04 (d, 1H, *J* = 8.0 Hz, Ar), 4.46 (br s, 2H, piperazinyl), 4.27 (s, 2H, SCH_2_), 4.10 (br s, 2H, piperazinyl), 3.37 (t, 4H, *J* = 4.0 Hz, piperazinyl), 2.80 (s, 3H, SMe). ^13^C NMR (*δ*): 197.4 (C=S), 166.4 (C=O), 154.2 (d, *J* = 243 Hz), 151.41, 126.3 (d, *J* = 10 Hz), 124.8 (d, *J* = 3 Hz), 121.8, 115.0 (d, *J* = 8 Hz), 45.6 (NCH_2_), 45.4 (NCH_2_), 40.8 (SMe), 35.4 (SCH_2_). HRMS: *m*/*z* [M]^+^ calculated for C_14_H_18_FN_3_O_3_S_3_: 391.0494; found: 391.0496. Analysis calculated for C_14_H_18_FN_3_O_3_S_3_: C, 42.95; H, 4.63; N, 10.73; found: C, 42.92; H, 4.67; N, 10.74%.

#### 3.2.8. 2-((4-Fluorophenyl)amino)-2-oxoethyl 4-(methylsulfonyl)piperazine-1-carbodithioate (**5f**)


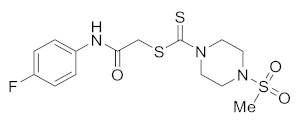


Yield 83%, m.p. 185–186 °C. IR: ν (cm^−1^): 3330 (NH), 1660 (C=O), 1520 (C=C), 1470 (CH_2_), 1222 (C=S). ^1^H NMR (*δ*): 8.80 (s, 1H, NH), 7.50 (m, 2H, Ar), 7.02 (m, 2H, Ar), 4.52 (br s, 2H, piperazinyl), 4.25 (s, 2H, SCH_2_), 4.09 (br s, 2H, piperazinyl), 3.40 (t, 4H, *J*= 4.0 Hz, piperazinyl), 2.85 (s, 3H, SMe). ^13^C NMR (*δ*): 197.4 (C=S), 168.3 (C=O),159.4 (d, *J* = 240 Hz),133.9, 121.6, 116.5, 50.0 (NCH_2_), 45.7 (NCH_2_), 40.3 (SMe), 35.3 (SCH_2_). HRMS: *m*/*z* [M]^+^ calculated for C_14_H_18_FN_3_O_3_S_3_: 391.0494; found 391.0496. Analysis calculated for C_14_H_18_FN_3_O_3_S_3_: C, 42.95; H, 4.63; N, 10.73; found: C, 42.97; H, 4.65; N, 10.76%.

#### 3.2.9. 2-((4-Methoxyphenyl)amino)-2-oxoethyl 4-(methylsulfonyl)piperazine-1-carbodithioate (**5g**)


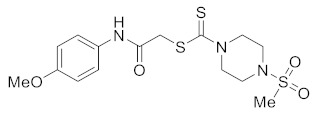


Yield 80%, m.p. 190 °C. IR: ν (cm^−1^): 3330 (NH), 1665 (C=O), 1528 (C=C), 1470 (CH_2_), 1222 (C=S). ^1^HNMR (*δ*): 8.63 (s, 1H, NH), 7.40 (d, 2H, *J* = 8.0 Hz, Ar), 6.83 (d, 2H, *J* = 8.0 Hz, Ar), 4.46 (br s, 2H, piperazinyl), 4.20 (s, 2H, SCH_2_), 4.09 (br s, 2H, piperazinyl), 3.76 (s, 3H, OMe), 3.36 (t, 4H, *J* = 4.0 Hz, piperazinyl), 2.80 (s, 3H, SMe). ^13^C NMR (*δ*): 197.4 (C=S), 166.1 (C=O), 157.0, 131.2, 122.3, 114.6, 56.0 (OMe), 50.2 (NCH_2_), 45.4 (NCH_2_), 40.7 (SMe), 35.8 (SCH_2_). HRMS: *m*/*z* [M]^+^ calculated for C_15_H_21_N_3_O_4_S_3_: 403.0694; found: 403.0697. Anal Calcd. for C_15_H_21_N_3_O_4_S_3_: C, 44.65; H, 5.25; N, 10.41; found: C, 44.67; H, 5.28; N, 10.45%.

#### 3.2.10. 2-((2,5-Dimethoxyphenyl)amino)-2-oxoethyl 4-(methylsulfonyl)piperazine-1-carbodithioate (**5h**)


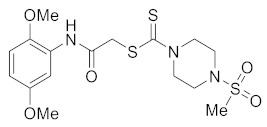


Yield 81%, m.p. 150 °C. IR: ν (cm^−1^): 3270 (NH), 1668 (C=O), 1524 (C=C), 1470 (CH_2_), 1222 (C=S). ^1^H NMR (*δ*): 8.87 (s, 1H, NH), 8.04 (s, 1H, Ar), 6.75 (d, 1H, *J* = 8.0 Hz, Ar), 6.54 (d, 1H, *J* = 8.0 Hz, Ar), 4.36 (br s, 2H, piperazinyl), 4.26 (s, 2H, SCH_2_), 4.16 (br s, 2H, piperazinyl), 3.81 (s, 3H, OMe), 3.75 (s, 3H, OMe), 3.35 (t, 4H, *J* = 4.0 Hz, piperazinyl), 2.79 (s, 3H, SMe). ^13^C NMR (*δ*): 196.6 (C=S), 165.9 (C=O), 153.9, 142.5, 128.3, 111.2, 109.0, 106.1, 56.6 (OMe), 55.8 (OMe), 45.3 (NCH_2_), 45.2 (NCH_2_), 41.3 (SMe), 35.2 (SCH_2_). HRMS: *m*/*z* [M]^+^ calculated for C_16_H_23_N_3_O_5_S_3_:433.0800; found: 433.0805. Analysis calculated for C_16_H_23_N_3_O_5_S_3_: C, 44.32; H, 5.35; N, 9.69; found: C, 44.35; H, 5.39; N, 9.73.

#### 3.2.11. 2-((2,4-Dimethylphenyl)amino)-2-oxoethyl 4-(methylsulfonyl)piperazine-1-carbodithioate (**5i**)


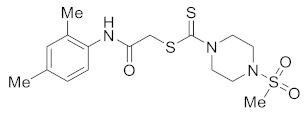


Yield 87%, m.p. 186–187 °C. IR: ν (cm^−1^): 3330 (NH), 1665 (C=O), 1525 (C=C), 1475 (CH_2_), 1220 (C=S). ^1^H NMR (*δ*): 8.30 (s, 1H, NH), 7.69 (d, 1H, *J* = 8.0 Hz, Ar), 6.99 (s, 1H, Ar), 6.96 (d, 1H, *J* = 8.0 Hz, Ar), 4.47 (br s, 2H, piperazinyl), 4.27 (s, 2H, SCH_2_), 4.13 (br s, 2H, piperazinyl), 3.35 (t, 4H, *J* = 4.0 Hz, piperazinyl), 2.80 (s, 3H, SMe), 2.26 (s, 3H, Me), 2.23 (s, 3H, Me). ^13^C NMR (*δ*): 197.4 (C=S), 166.6 (C=O), 135.0, 133.2, 131.3, 129.2, 127.3, 122.8, 45.8 (NCH_2_) 45.4 (NCH_2_), 40.6 (SMe), 35.4 (SCH_2_), 21.0 (Me), 18.4 (Me). HRMS: *m*/*z* [M]^+^ calculated for C_16_H_23_N_3_O_3_S_3_: 401.0902; found: 401.0906. Analysis calculated for C_16_H_23_N_3_O_3_S_3_: C, 47.86; H, 5.77; N, 10.46; found: C, 47.90; H, 5.79; N, 10.48%.

#### 3.2.12. 2-((3,4-Dimethylphenyl)amino)-2-oxoethyl-4-(methylsulfonyl)piperazine-1-carbodithioate (**5j**)


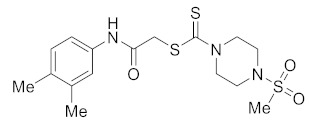


Yield 82%, m.p. 170–171 °C. IR: ν (cm^−1^): 3235 (NH), 1660 (C=O), 1525 (C=C), 1470 (CH_2_), 1220 (C=S). ^1^H NMR (*δ*): 8.65 (s, 1H, NH), 7.28 (s, 1H, Ar), 7.26 (d, 1H, *J* = 8.0 Hz, Ar), 7.08 (d, 1H, *J* = 8.0 Hz, Ar), 4.53 (br s, 2H, piperazinyl), 4.26 (s, 2H, SCH_2_), 4.15 (br s, 2H, piperazinyl), 3.39 (t, 4H, *J* = 4.0 Hz, piperazinyl), 2.84 (s, 3H, SMe), 2.26 (s, 3H, Me), 2.23 (s, 3H, Me). ^13^C NMR (*δ*): 197.4 (C=S), 163.9 (C=O), 137.9, 135.9, 133.2, 129.9, 121.9, 117.1, 50.0 (NCH_2_), 49.6 (NCH_2_), 40.9 (SMe), 35.2 (SCH_2_) 19.9 (Me), 19.2 (Me). HRMS: *m*/*z* [M]^+^ calculated for C_16_H_23_N_3_O_3_S_3_: 401.0902; found: 401.0906. Analysis calculated for C_16_H_23_N_3_O_3_S_3_: C, 47.86; H, 5.77; N, 10.46; found: C, 47.89; H, 5.77; N, 10.50%.

### 3.3. Experimental Procedures for Antiproliferative, Hemolytic, and Thrombolytic Activity

#### 3.3.1. Culture and Treatment of Cell Lines

The activity of piperazine-1-carbodithioates **5a**–**5j** against human liver carcinoma (A-549) was investigated. Dulbecco’s Modified Eagles Medium (DMEM) was used for the cultural growth of A-549. The DMEM was enriched with fetal bovine serum (10%), streptomycin (100 units/mL), and penicillin (100 μg/mL). The inoculation was carried out in a moistened atmosphere containing carbon dioxide (5%). Compounds **5a**–**5j** were dissolved in dimethyl sulfoxide (DMSO). The cell cultures (0.05%) were added to DMSO and employed as a negative control [32,33].

#### 3.3.2. Evaluation of Cell Viability

A-549 cells (100 µL) were cultured overnight in 96-well plates, treated with compounds **5a**–**5j**, and incubated (48 h at 37 °C). The MTT reagent (10 µL, 5 mg/mL) was added to each plate and incubated (4 h at 37 °C). In the final step, each cell plate was diluted with DMSO (150 µL), and the absorbance was estimated at 490 nm using a microplate-reader to calculate the cell viability percentage.

#### 3.3.3. Hemolytic Potential

A blood sample (5 mL) from albino mice was centrifuged (1000 rpm for 5 min). The pellet of red blood cells (RBCs) was isolated and washed four times with chilled phosphate buffer saline (pH 7.4). A solution of **5a**–**5j** (20 μL; 10 mg/mL) was added to the RBCs pellet (180 μL). The sample was incubated (30 min at 37 °C); removed from the incubator; cooled in an ice bath for 5 min; and centrifuged (13,000 rpm for 5 min). Ice-cold phosphate buffer saline was added to the supernatant (100 µL) in each tube. The DMSO was designated as a negative control, while 2,2′-azino-*bis* (3-ethylbenzothiazoline-6-sulfonic acid (ABTS)) served as a positive control. The absorbance of the sample was measured at 517 nm, and the age hemolysis (%) was calculated using Equation (1) [34].

(1) $$\mathrm{age}\;\mathrm{hemolysis}\;\left(\%\right)=\frac{\mathrm{Absorbance}\;\mathrm{of}\;\mathrm{sample}-\mathrm{Absorbance}\;\mathrm{of}\;\mathrm{negative}\;\mathrm{control}}{\mathrm{Absorbance}\;\mathrm{of}\;\mathrm{positive}\;\mathrm{control}}\times 100$$

#### 3.3.4. Thrombolytic Potential

A blood sample (1 mL) was taken from albino mice and transferred to various rinsed and weighed Eppendorf tubes. The Eppendorf tube sample was incubated (1 h at 37 °C) to form clots. The serum was discarded, and the Eppendorf tubes were weighed to calculate the initial weight of the clot. A solution (100 μL) of **5a**–**5j** in DMSO was added separately to the Eppendorf tubes and incubated (3 h at 37 °C). The ABTS was used as a positive control, while water was used as a negative control. The serum was removed again, and the Eppendorf tubes were weighed to measure the clot lysis (%) using Equation (2) [35].

(2) $$\mathrm{Clot}\;\mathrm{lysis}\;\left(\%\right)=\frac{\mathrm{Initial}\;\mathrm{clot}\;\mathrm{weight}-\mathrm{Final}\;\mathrm{clot}\;\mathrm{weight}}{\mathrm{Initial}\;\mathrm{weight}\;\mathrm{of}\;\mathrm{the}\;\mathrm{clot}}\times 100$$

### 3.4. Computational Study

Compound **5b** was investigated for its in silico-modeled anticancer activity and potential mechanism of action. A potential target of compound **5b** was predicted using the SwissTargetPrediction tool. It employs a ligand-based approach and derives the probability of prediction using 2D and 3D similarity to chemical compounds with known targets [36]. Compound **5b** was molecularly docked on a predicted target to investigate its binding affinity, mode of action, and interactions using the induced fit docking (IFD) protocol in the Molecular Operating Environment (MOE) 2015.10. The IFD protocol was validated by cognate redocking of the co-crystalized ligand within the active site, with the root-mean-square deviation (RMSD) serving as the measure of validation. The PubChem CID: 11704101 (PQ-10) is an inhibitor of PDE10A in lung carcinoma and is modeled as a standard molecule in this simulation. The 3D X-ray crystalized structure of phosphodiesterase 10A (PDB ID: 3HR1; 1.53 Å) was retrieved from the RSCB Protein Data Bank (http://www.rscb.org; accessed on 2 February 2022). The inherent structural problems of macromolecules were corrected in the QuickPrep module of MOE. The structure was further protonated and minimized under the Amber 10:EHT forcefield to optimize the molecular mechanics’ refinements and tether restraints in docked poses. The Site Finder module defined the binding pocket in the vicinity of the co-crystallized ligand at the active site. The Dock module was used to dock Compound **5b** using the triangular matcher placement method and the London dG scoring function. The docked poses were further refined using the IFD method and the GBVI/WSA dG scoring function. A pose with the lowest binding free energy (ΔG) was investigated further for potential binding affinity, binding pose, and interactions using Discovery Studio Visualizer v17.2 (San Diego, CA, USA).

## 4. Conclusions

The structures of a new class of *N*-methylsulfonyl-piperazine-based acetamides have been determined. Their anticancer potential against human lung carcinoma (A-549) was investigated. The types of substituents on the aryl ring within the synthesized piperazines affect the anticancer activity. For example, piperazines containing 2-chlorophenyl and 2,4-dichlorophenyl moieties showed the most potent antiproliferative activity. Piperazines with 2,4-dichlorophenyl and 3,4-dichlorophenyl rings showed the lowest cytotoxicity. The cytotoxic effect of the synthesized piperazines was investigated through hemolysis and thrombolysis. The piperazine with a 2,5-dimethoxylphenyl moiety had the greatest thrombolytic effect. The anticancer activity of the most potent piperazine was investigated further using in silico studies to delineate its potential mechanism of action. More structural modifications to piperazine aryl rings are required to create more potent and selective antiproliferative and cytotoxic candidates.

## Figures and Tables

**Table 1:** The anti-lung cancer activity, hemolytic, and thrombolytic potential of piperazine-1-carbodithioates **5a**–**5j** according to Scheme 1.

Piperazine	Ar	Cell Viability A-549 ^a^	Hemolysis (%)	Thrombolysis (%)
**5a**	Phenyl	61.35 ± 2.29	3.1	58.9
**5b**	2-Chlorophenyl	25.11 ± 2.49	1.27	54.4
**5c**	4-Chlorophenyl	60.29 ± 5.96	10	47.8
**5d**	3,4-Dichlorophenyl	38.08 ± 2.85	0.1	56.03
**5e**	2-Fluorophenyl	68.94 ± 6.64	0.3	53.5
**5f**	4-Fluorophenyl	41.01 ± 3.73	2.8	50.3
**5g**	4-Methoxyphenyl	43.36 ± 4.42	1.3	50.9
**5h**	2,5-Dimethoxyphenyl	40.25 ± 3.34	7.1	60.2
**5i**	2,4-Dimethylphenyl	25.31 ± 3.62	2.8	57.4
**5j**	3,4-dimethylphenyl	47.94 ± 1.57	0.1	58.7
Standard		100 ± 0		
DMSO			0.01	0.57
ABTS			95.9	80

^a^ Cell viability: (Mean ± SD) in triplicate with a concentration of 100 μg/mL. DMSO and ABTS were used as negative and positive controls, respectively.

**Table 2:** The IFD parameters of **5b** and PQ-10 simulated in the active site of PDE10A.

Ligand	ΔG (kcal/mol)	Interacting Residues	Interaction Type
**5b**	−8.30	TYR514, PHE686, TYR683, VAL712, PRO702, MET703, GLU711, SER667	H–bonding, sulfur–X, π–σ, π–π stacked, amide–π stacked, alkyl, π–alkyl
PQ-10	−8.97	GLU711, PRO702, TYR683, VAL712, GLN716, ILE682, ALA679, TYR514, ASP664, PHE716, MET703	H–bonding, sulfur–X, π–σ, π–π stacked, amide–π stacked, alkyl, π–alkyl

## Data Availability

Data are contained within the article.

## Supplementary Materials

The following supporting information can be downloaded at: https://www.mdpi.com/article/10.3390/molecules27154776/s1, Figure S1: ^1^H NMR spectrum of **5a**, Figure S2: ^13^C NMR spectrum of **5a**, Figure S3: ^1^H NMR spectrum of **5b**, Figure S4: ^13^C NMR spectrum of **5b**, Figure S5: ^1^H NMR spectrum of **5c**, Figure S6: ^13^C NMR spectrum of **5c**, Figure S7: ^1^H NMR spectrum of **5d**, Figure S8: ^13^C NMR spectrum of **5d**, Figure S9: ^1^H NMR spectrum of **5e**, Figure S10: ^13^C NMR spectrum of **5e**, Figure S11: ^1^H NMR spectrum of **5f**, Figure S12: ^13^C NMR spectrum of **5f**, Figure S13: ^1^H NMR spectrum of **5g**, Figure S14: ^13^C NMR spectrum of **5g**, Figure S15: ^1^H NMR spectrum of **5h**, Figure S16: ^13^C NMR spectrum of **5h**, Figure S17: ^1^H NMR spectrum of **5i**, Figure S18: ^13^C NMR spectrum of **5i**, Figure S19: ^1^H NMR spectrum of **5j**, and Figure S20: ^13^C NMR spectrum of **5j**.

## Sample Availability

Samples of compounds **5** are available from the authors.
