# Supplementary material for: Ultrasound-Assisted Synthesis and In Silico Modeling of Methanesulfonyl-Piperazine-Based Dithiocarbamates as Potential Anticancer, Thrombolytic, and Hemolytic Structural Motifs
Source: Molecules. 2022 Jul 26;27(15):4776. doi: 10.3390/molecules27154776 (PMC9369641; doi:10.3390/molecules27154776)
Supplement: Supplementary file 1 [file molecules-27-04776-s001.zip › molecules-1821672-supplementary.pdf]

molecules

MDPI

Supplementary Materials

Ultrasound-Assisted Synthesis and *In-Silico* Modeling of Methanesulfonyl-Piperazine-Based Dithiocarbamates as Potential Anticancer, Thrombolytic, and Hemolytic Structural Motifs

Freeha Hafeez [1], Ameer Fawad Zahoor [1,*], Azhar Rasul [2], Asim Mansha [1], Razia Noreen [3], Zohaib Raza [4], Kulsoom Ghulam Ali [1], Ali Irfan [1] and Gamal A. El-Hiti [5,*]

[1] Department of Chemistry, Government College University Faisalabad, Faisalabad 38000, Pakistan; fariha5882@gmail.com (F.H.); mansha.asim@gmail.com (A.M.); kulsoom_gcu@yahoo.com (K.G.A.); raialiirfan@gmail.com (A.I.)

[2] Department of Zoology, Government College University Faisalabad, Faisalabad 38000, Pakistan; drazharrasul@gmail.com

[3] Department of Biochemistry, Government College University Faisalabad, Faisalabad 38000, Pakistan; razianoreen@hotmail.com

[4] Department of Pharmacology, Government College University Faisalabad, Faisalabad 38000, Pakistan; zuhaib.raza@live.com

[5] Cornea Research Chair, Department of Optometry, College of Applied Medical Sciences, King Saud University, Riyadh 11433, Saudi Arabia

* Correspondence: fawad.zahoor@gcuf.edu.pk (A.F.Z.); gelhiti@ksu.edu.sa (G.A.E.-H.); Tel.: +966-114693778 (G.A.E.-H.); Fax: +966-114693536 (G.A.E.-H.)

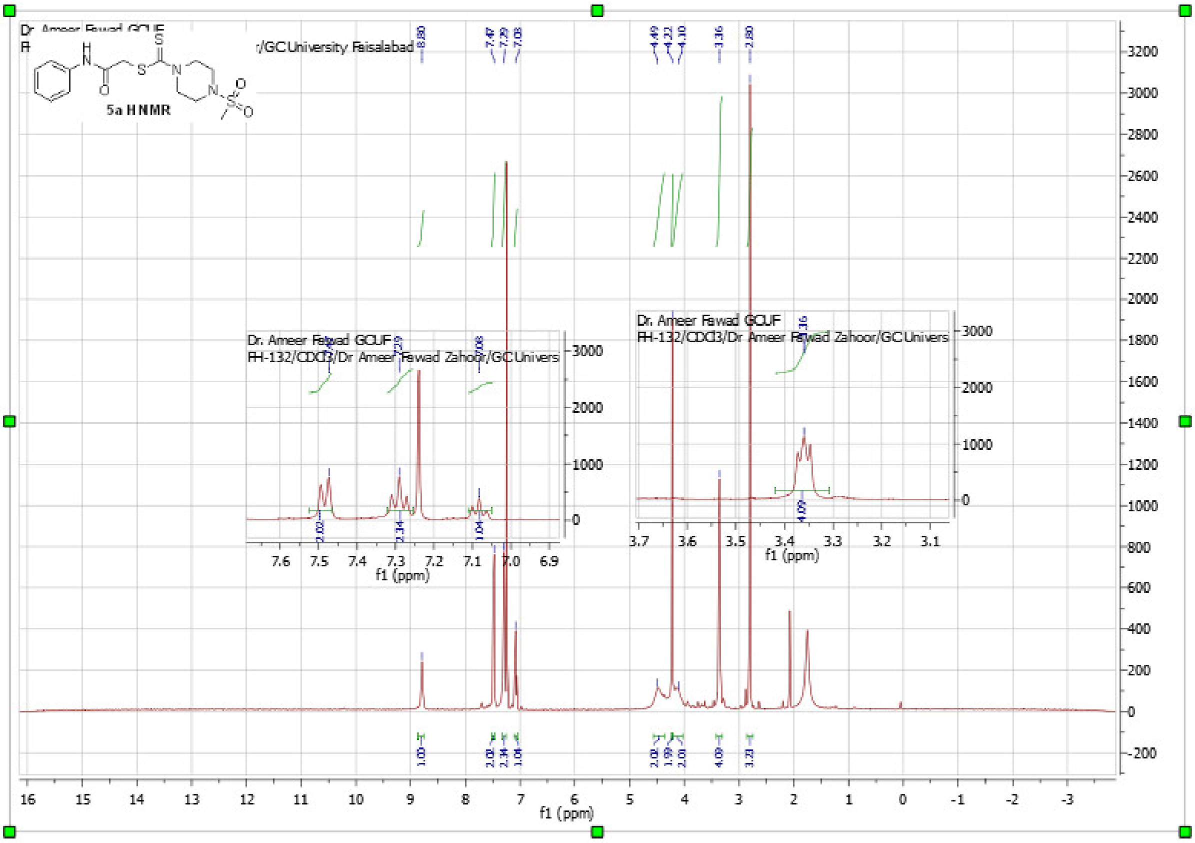

Figure S1. ^{1}H NMR of compound **5a**.

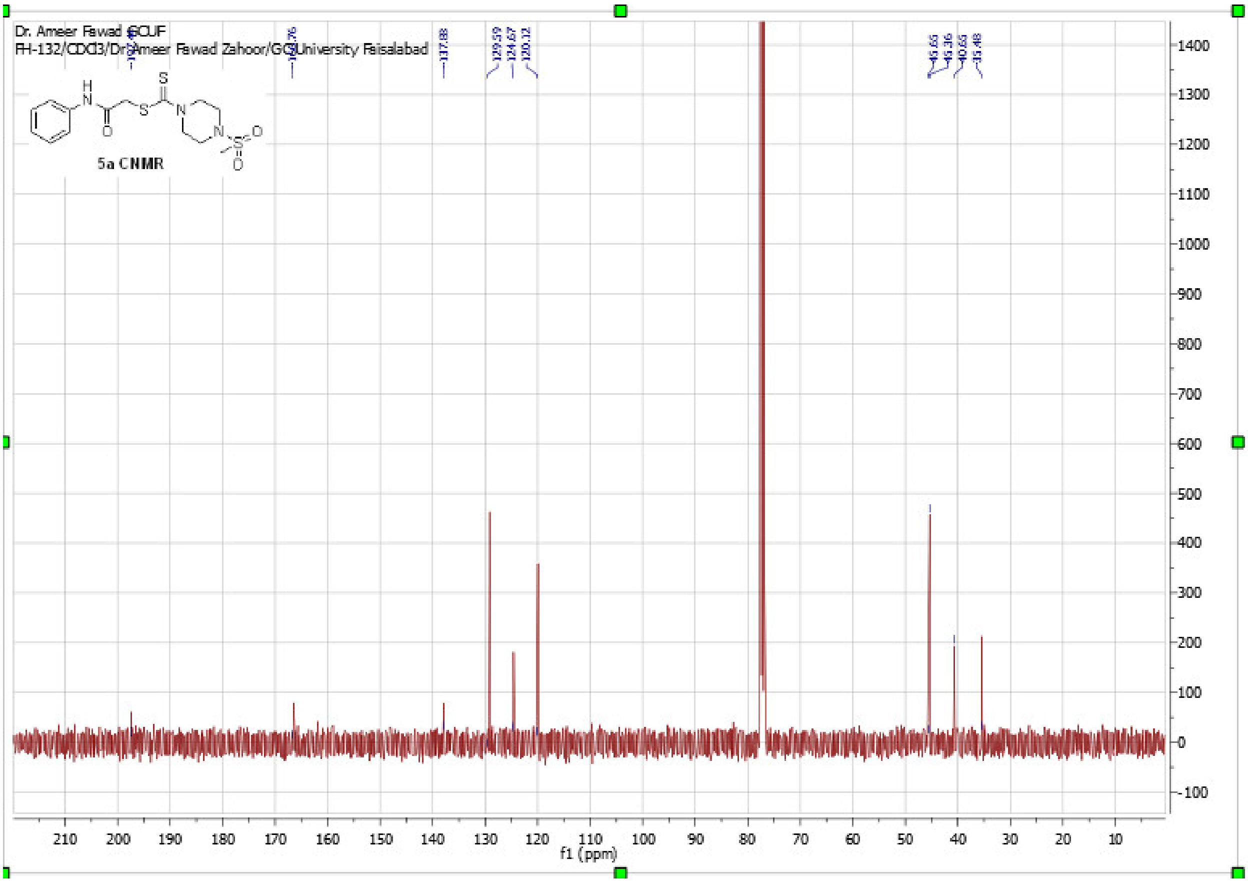

Figure S2. ^{13}C NMR of compound **5a**.

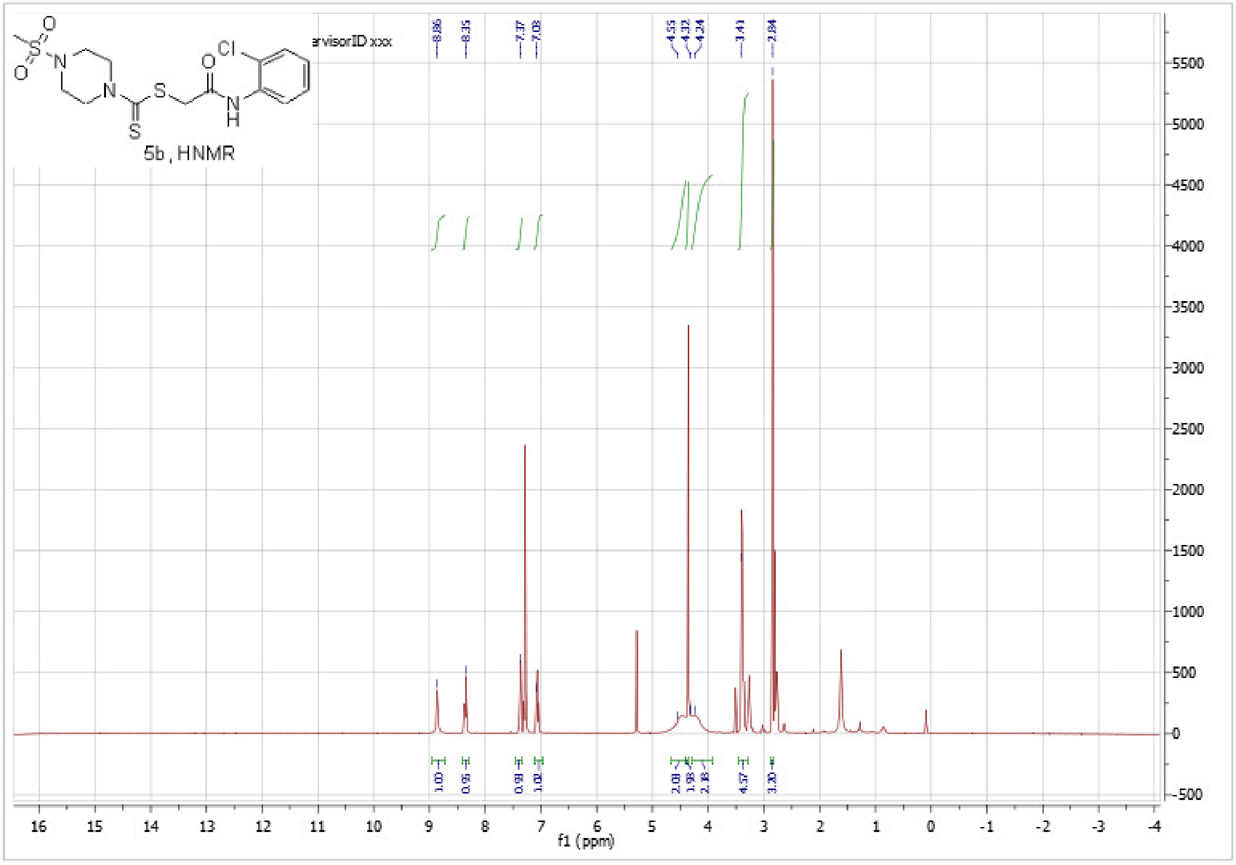

Figure S3. ^{1}H NMR of compound **5b**.

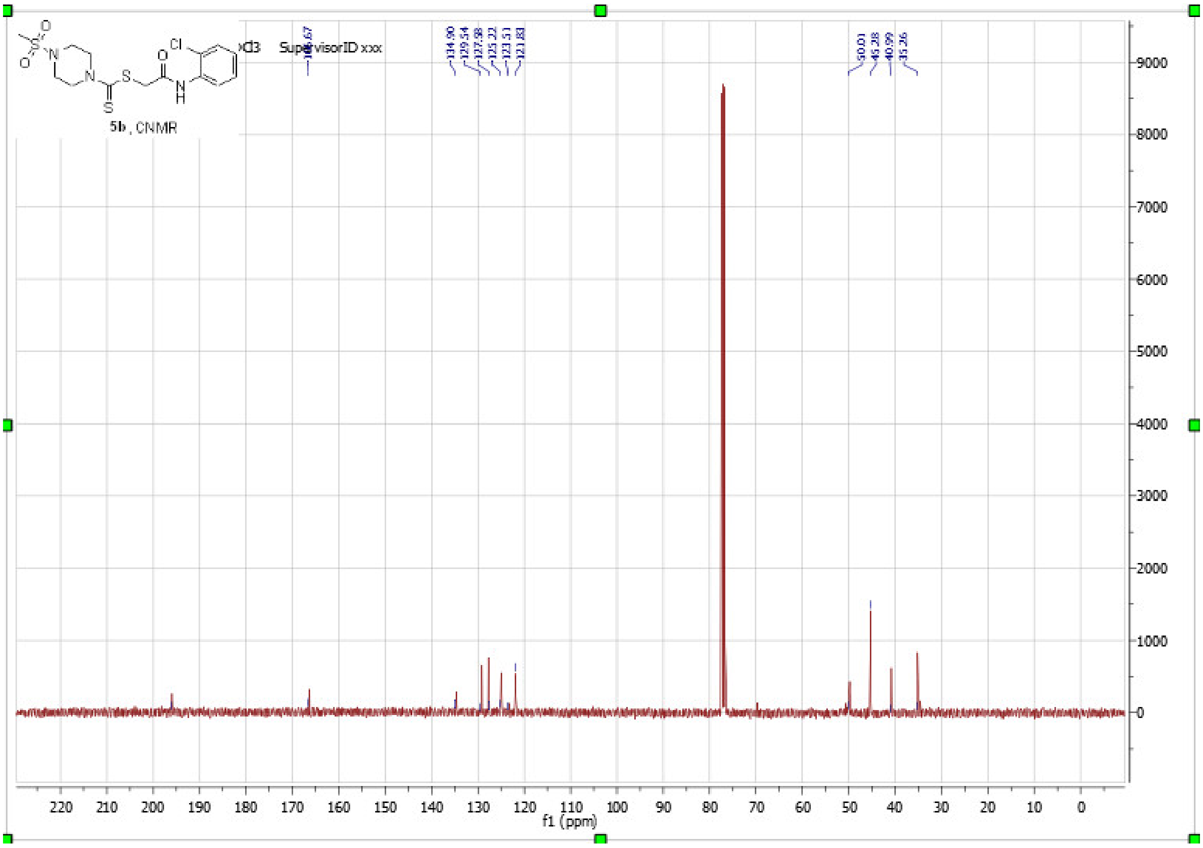

Figure S4. ^{13}C NMR of compound **5b**.

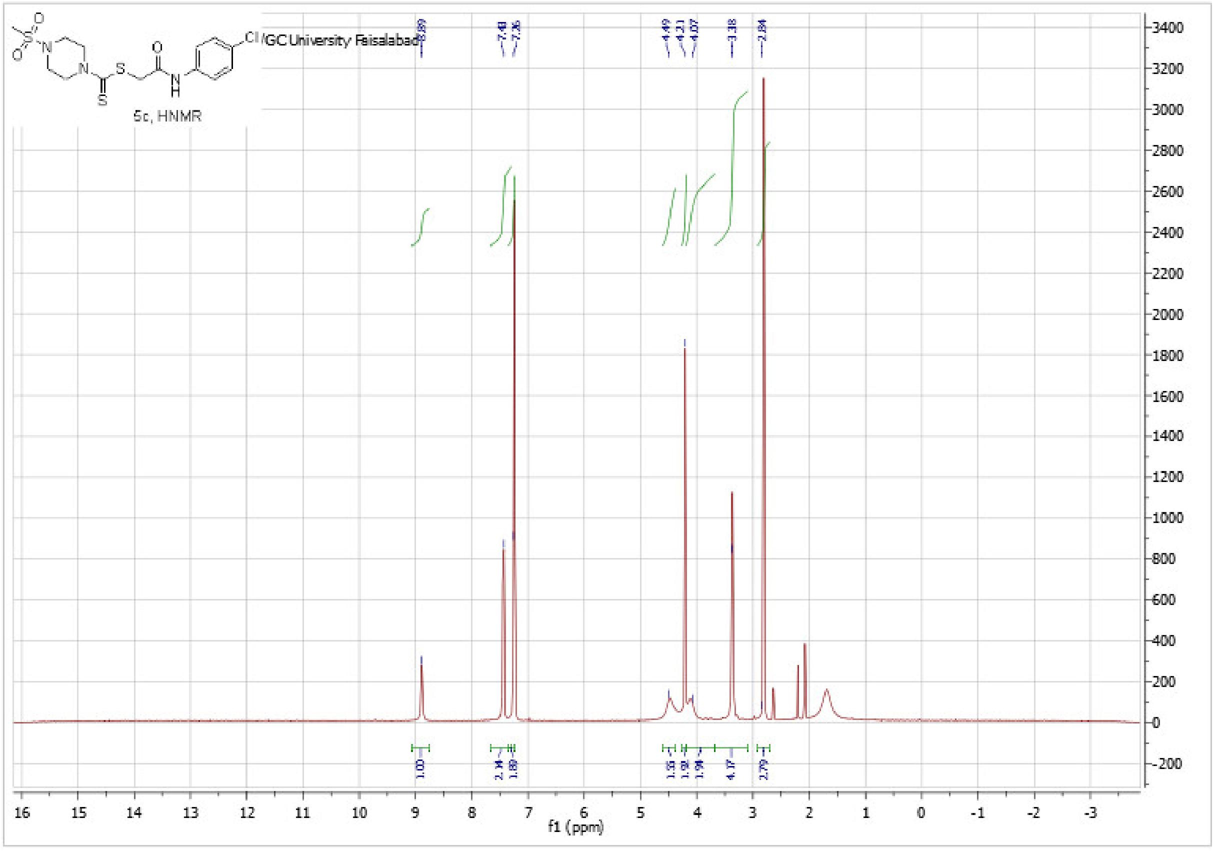

Figure S5. ^{1}H NMR of compound **5c**.

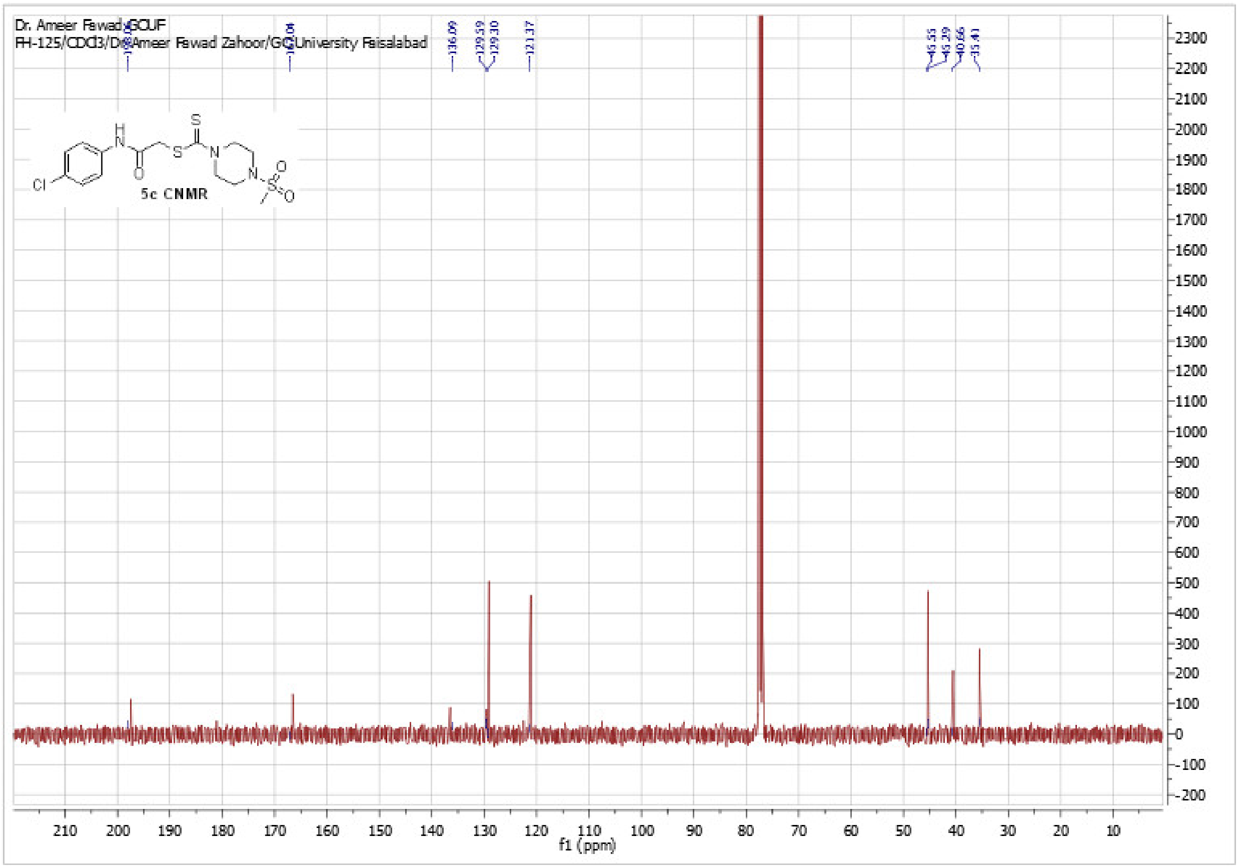

Figure S6. ^{13}C NMR of compound **5c**.

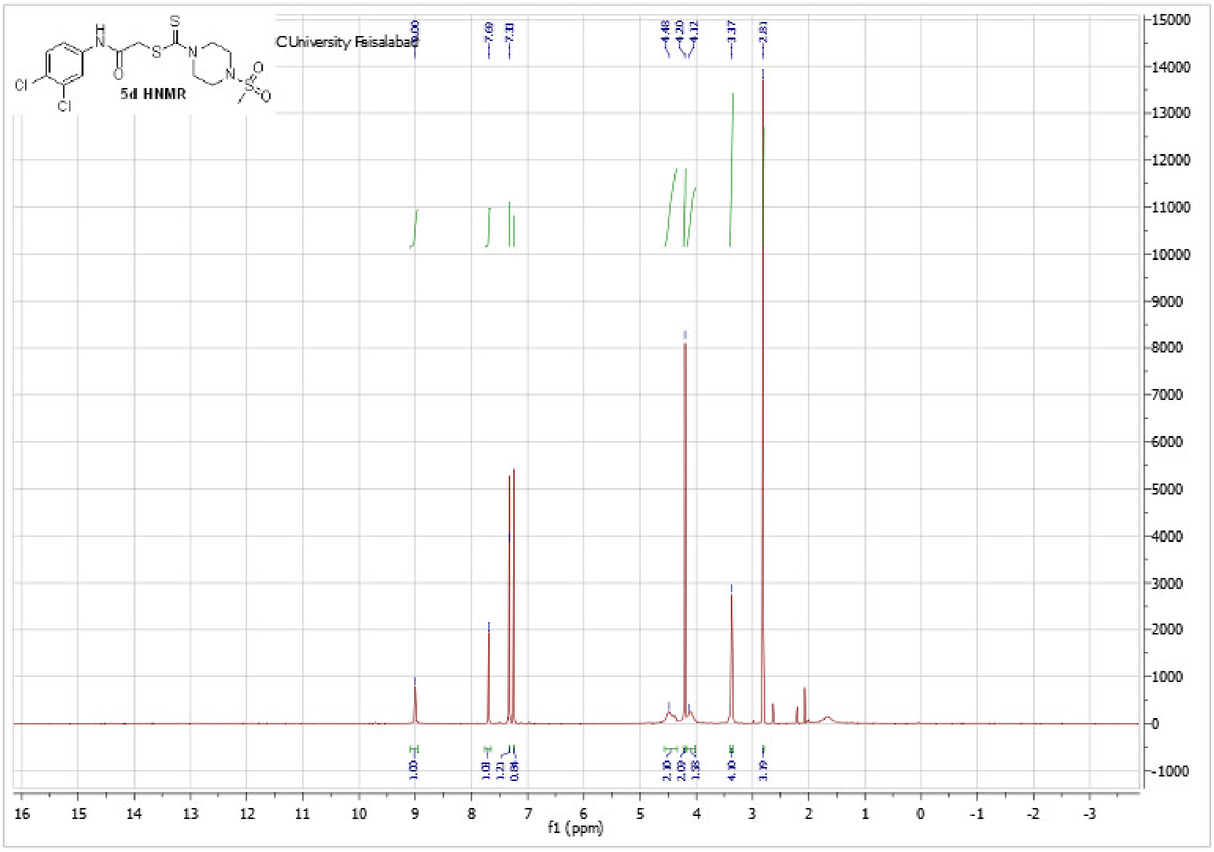

Figure S7. ^{1}H NMR of compound **5d**.

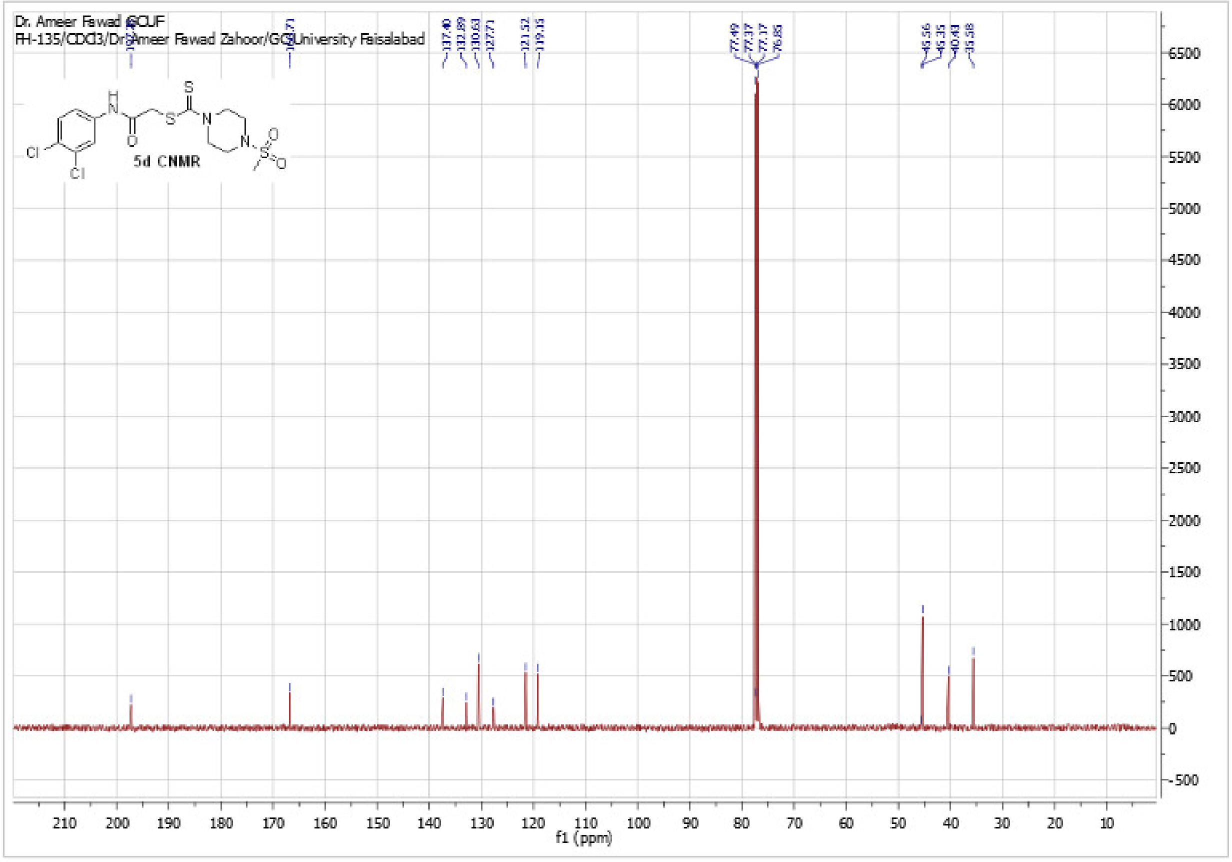

Figure S8: ^{13}C NMR of compound **5d**.

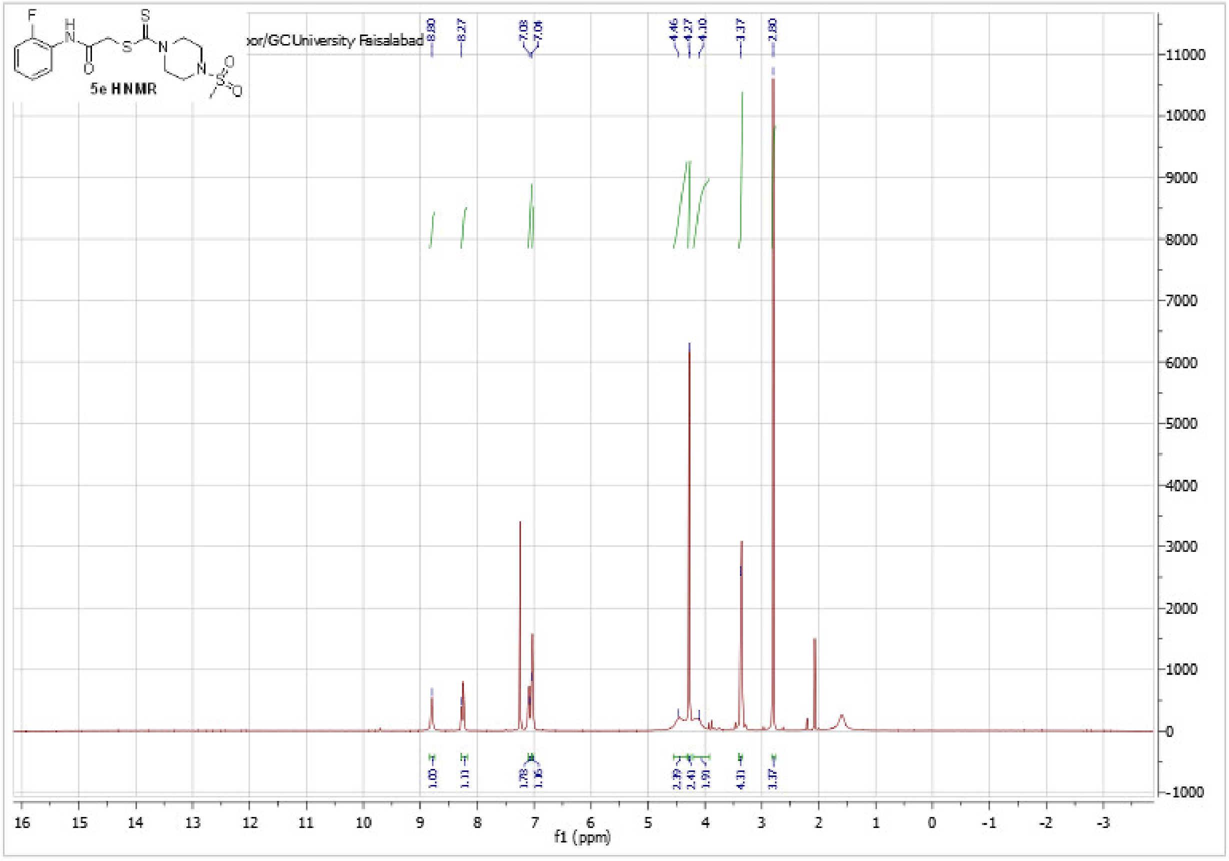

Figure S9. ^{1}H NMR of compound **5e**.

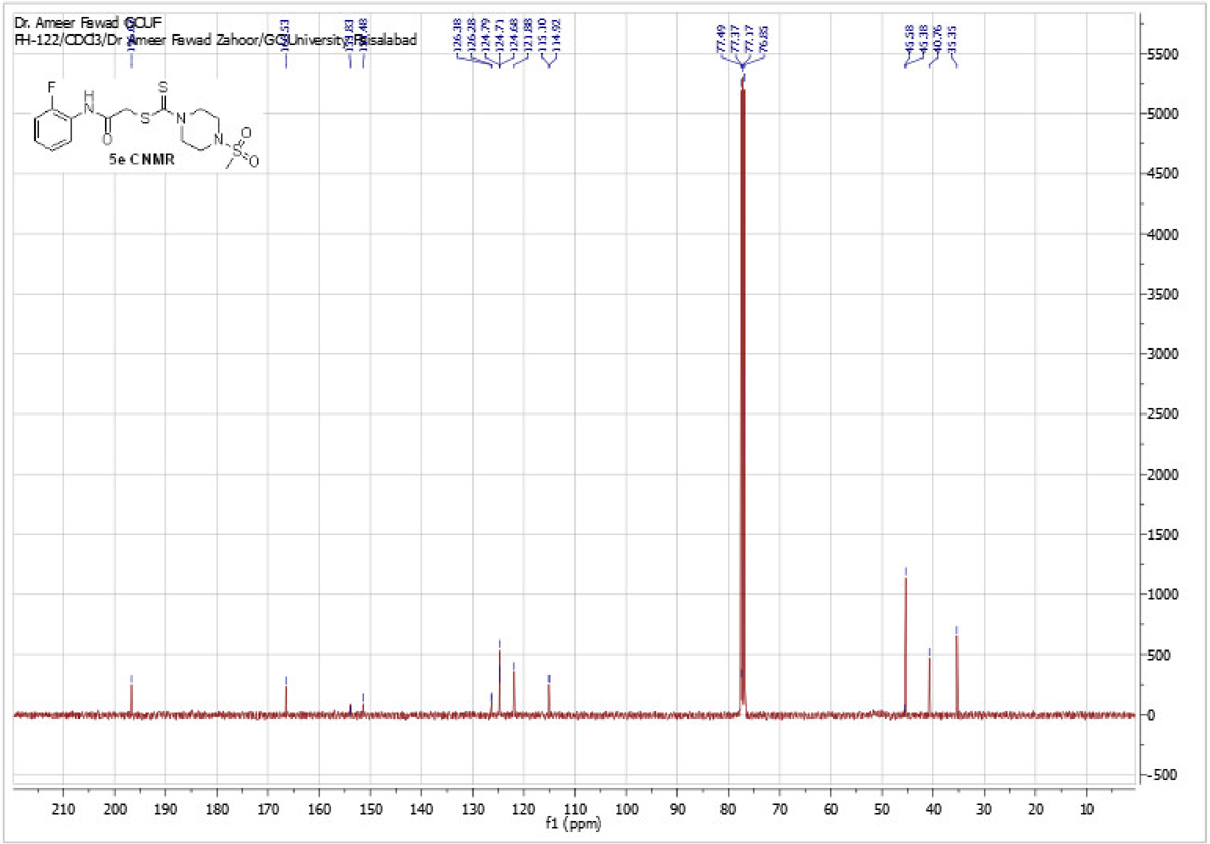

Figure S10. ^{13}C NMR of compound **5e**.

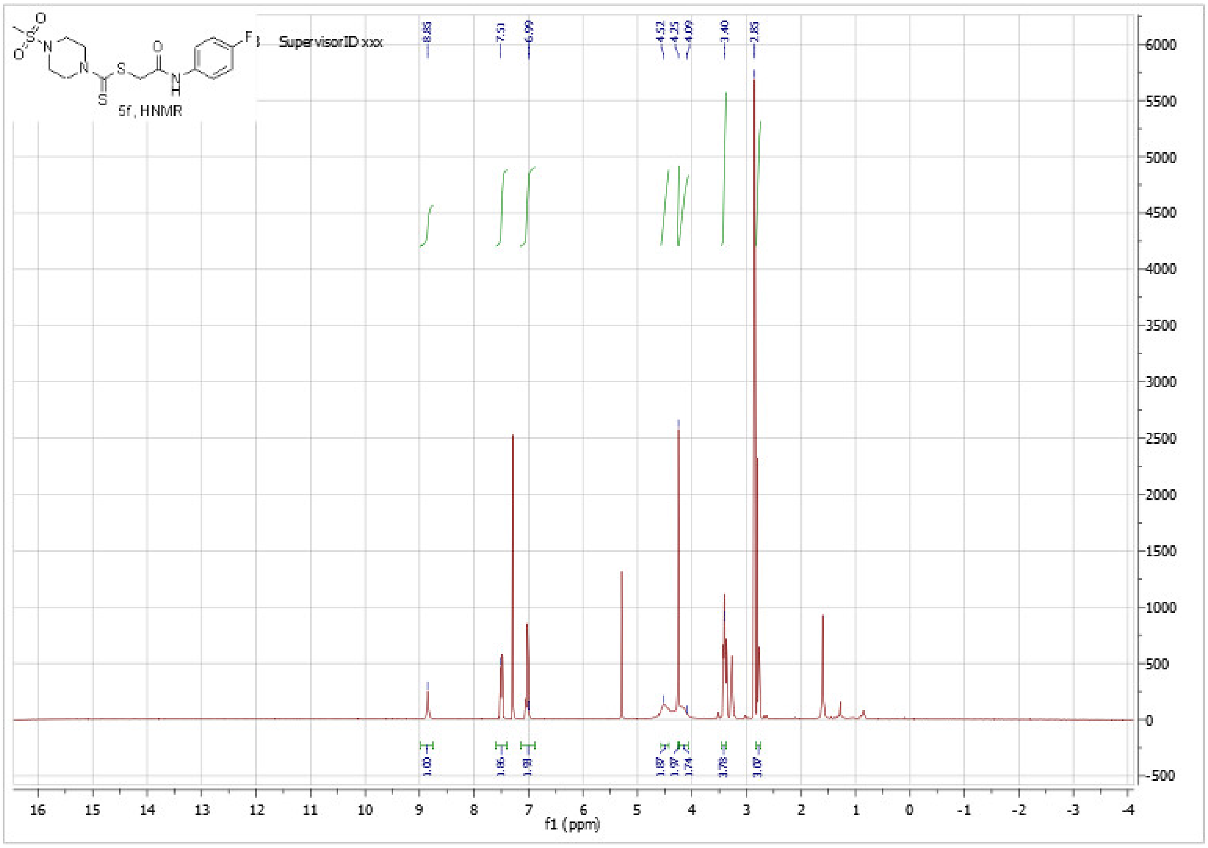

Figure S11. ^{1}H NMR of compound **5f**.

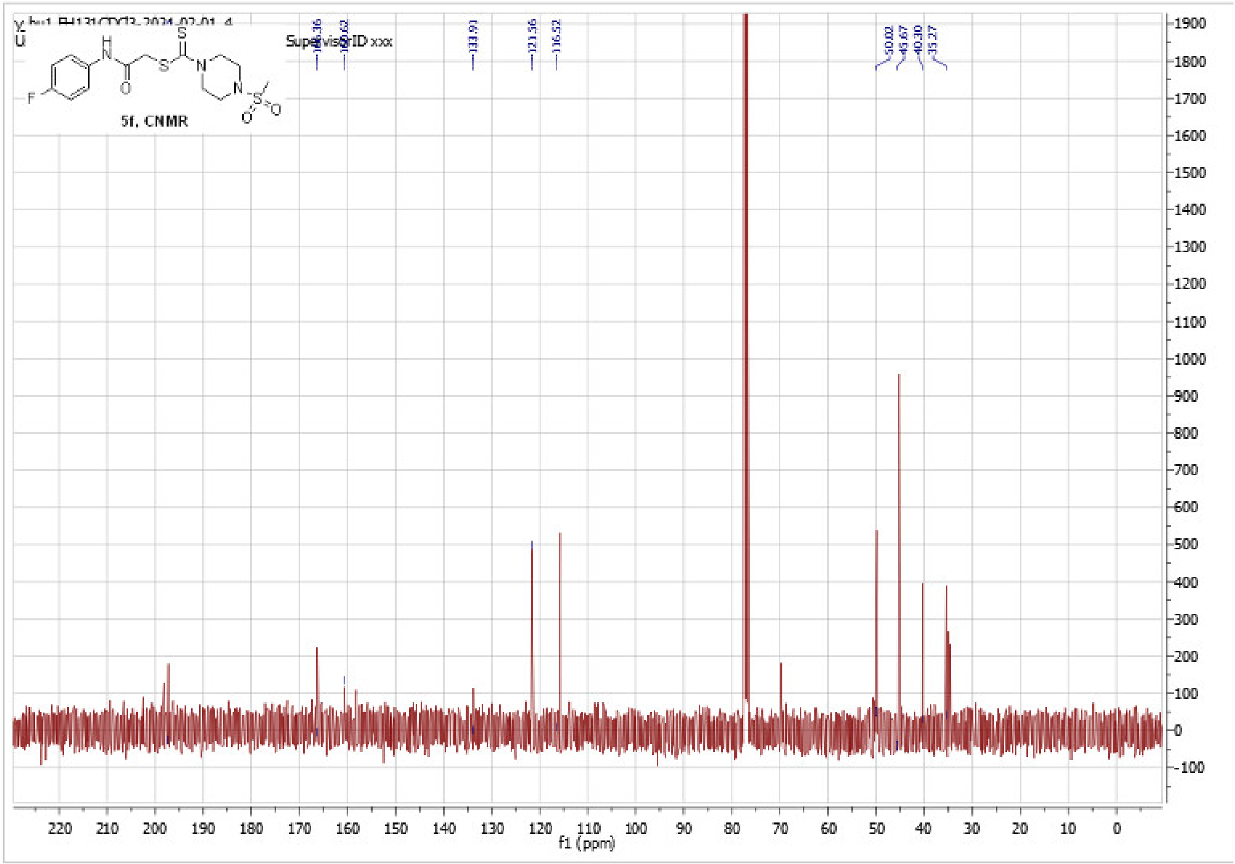

Figure S12. ^{13}C NMR of compound **5f**.

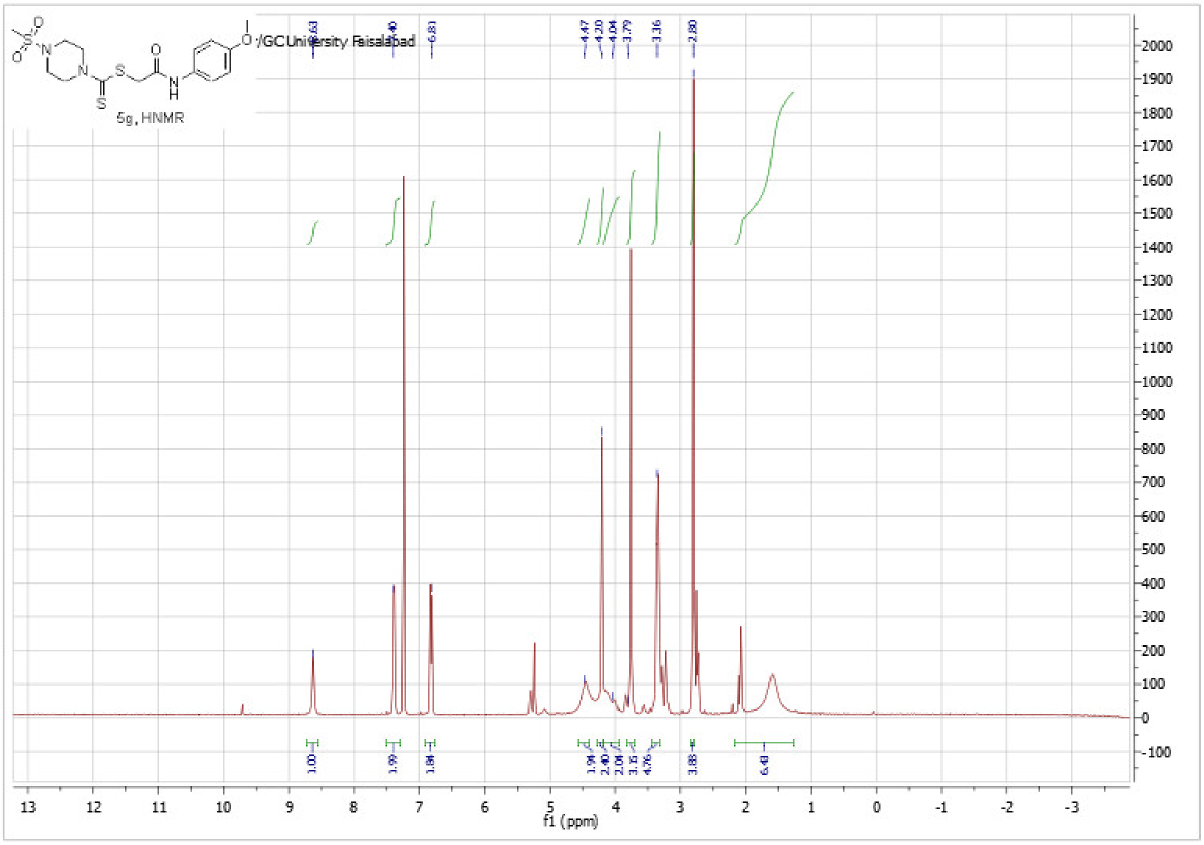

Figure S13. ^{1}H NMR of compound **5g**.

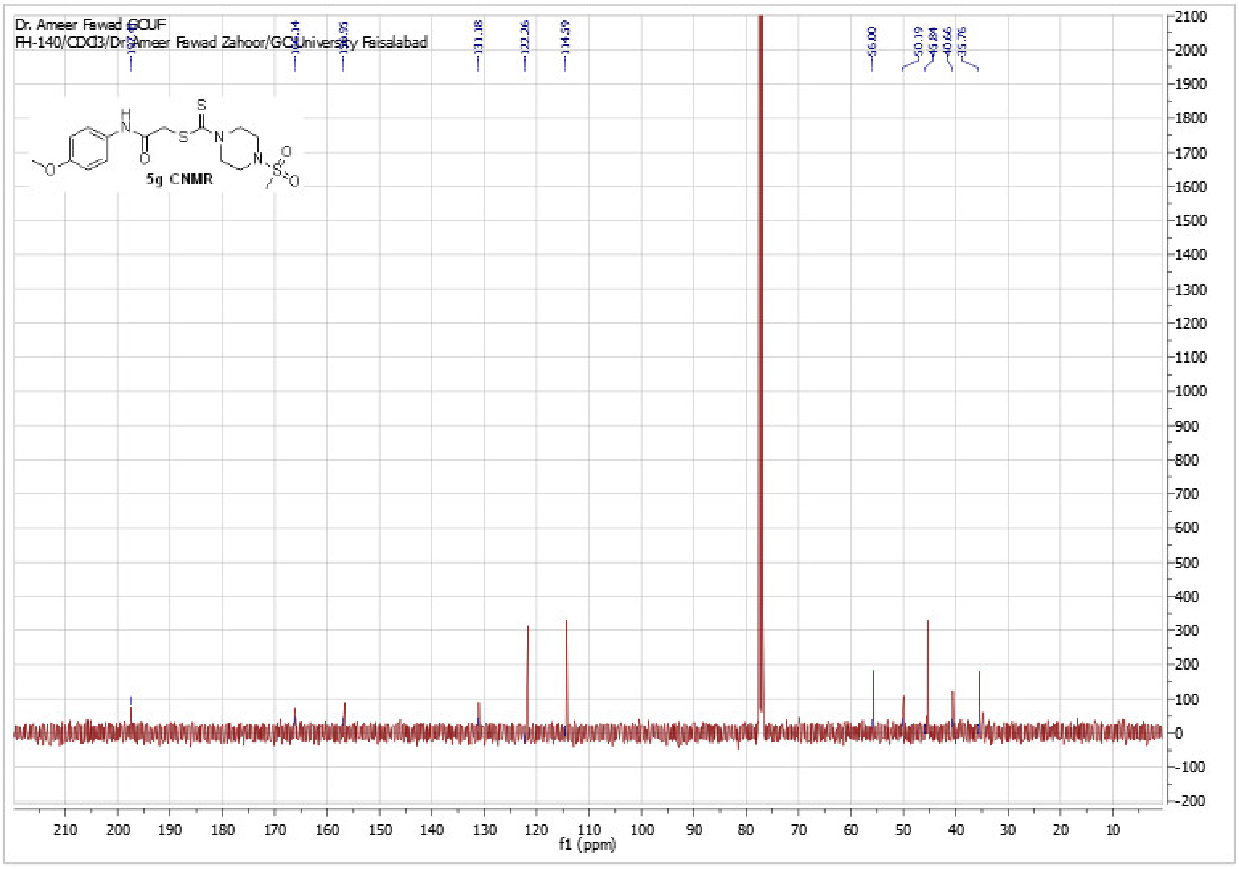

Figure S14. ^{13}C NMR of compound **5g**.

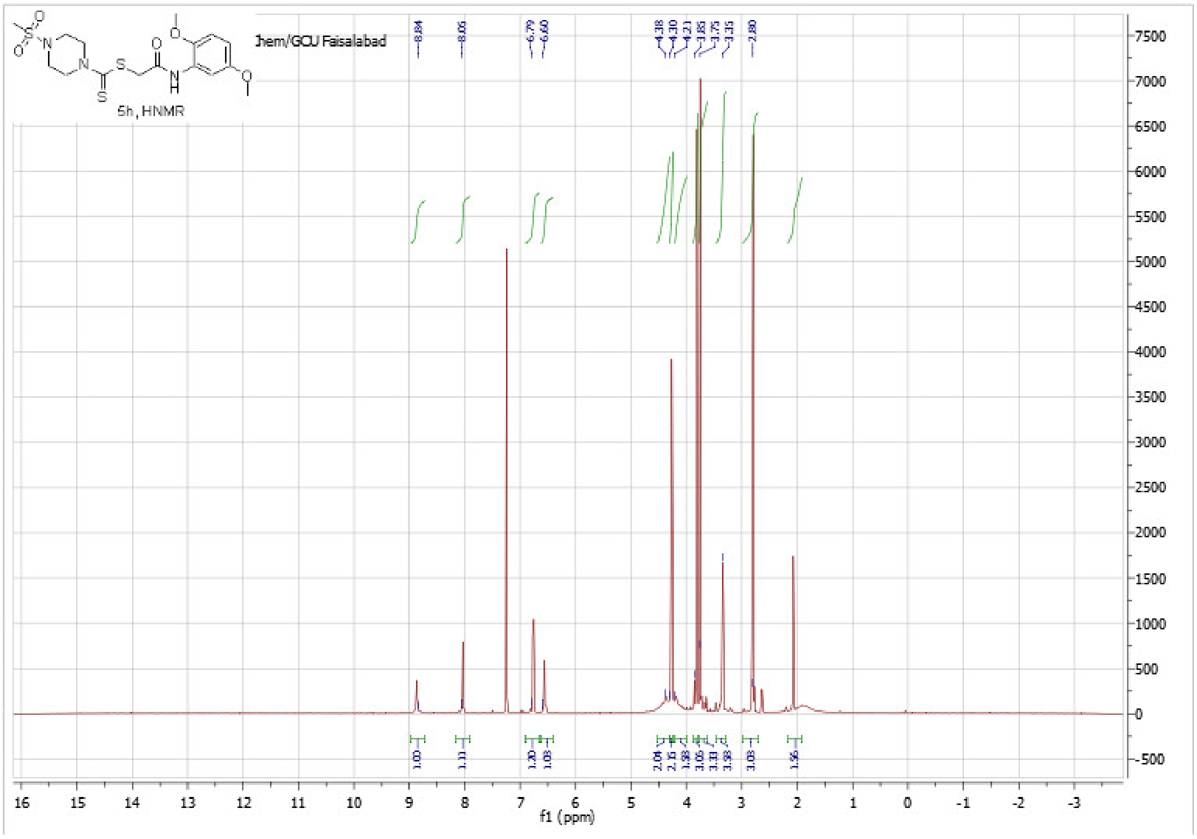

Figure S15. ^{1}H NMR of compound **5h**.

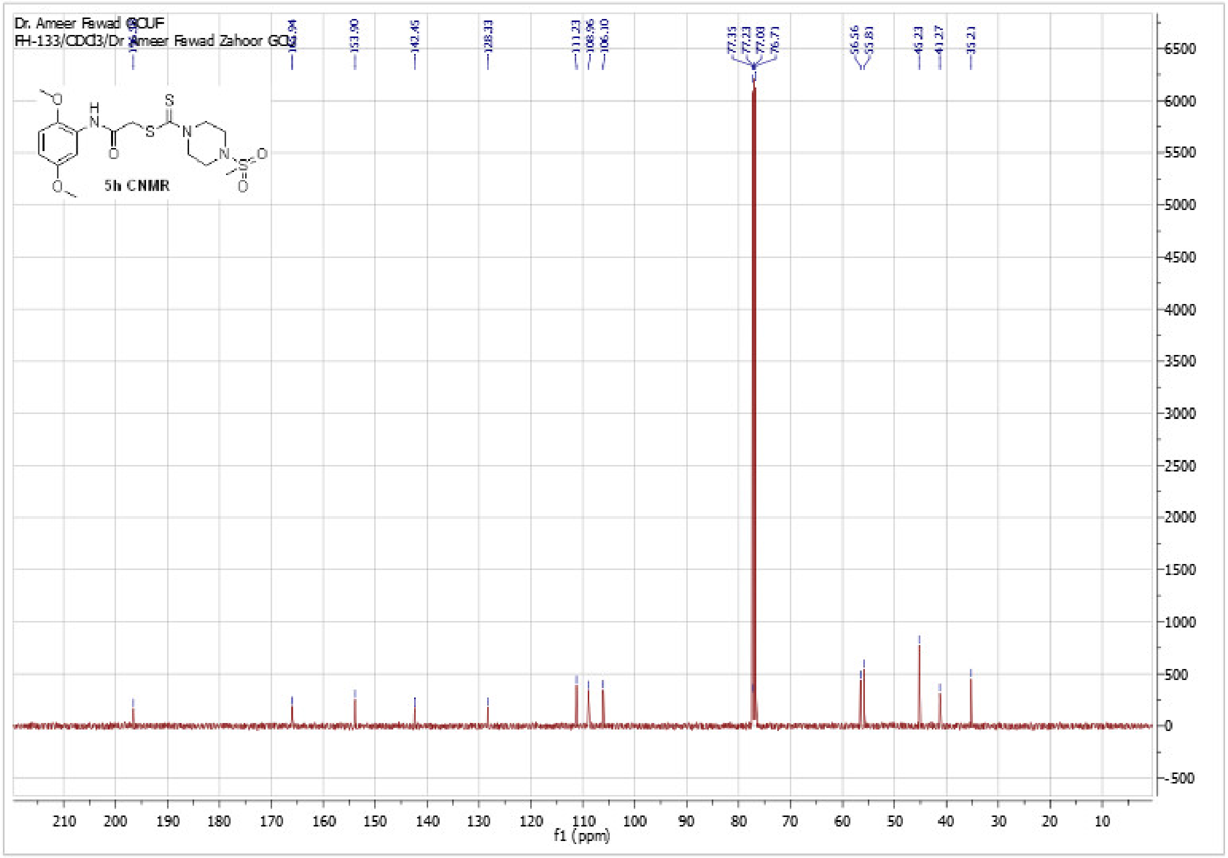

Figure S16. ^{13}C NMR of compound **5h**.

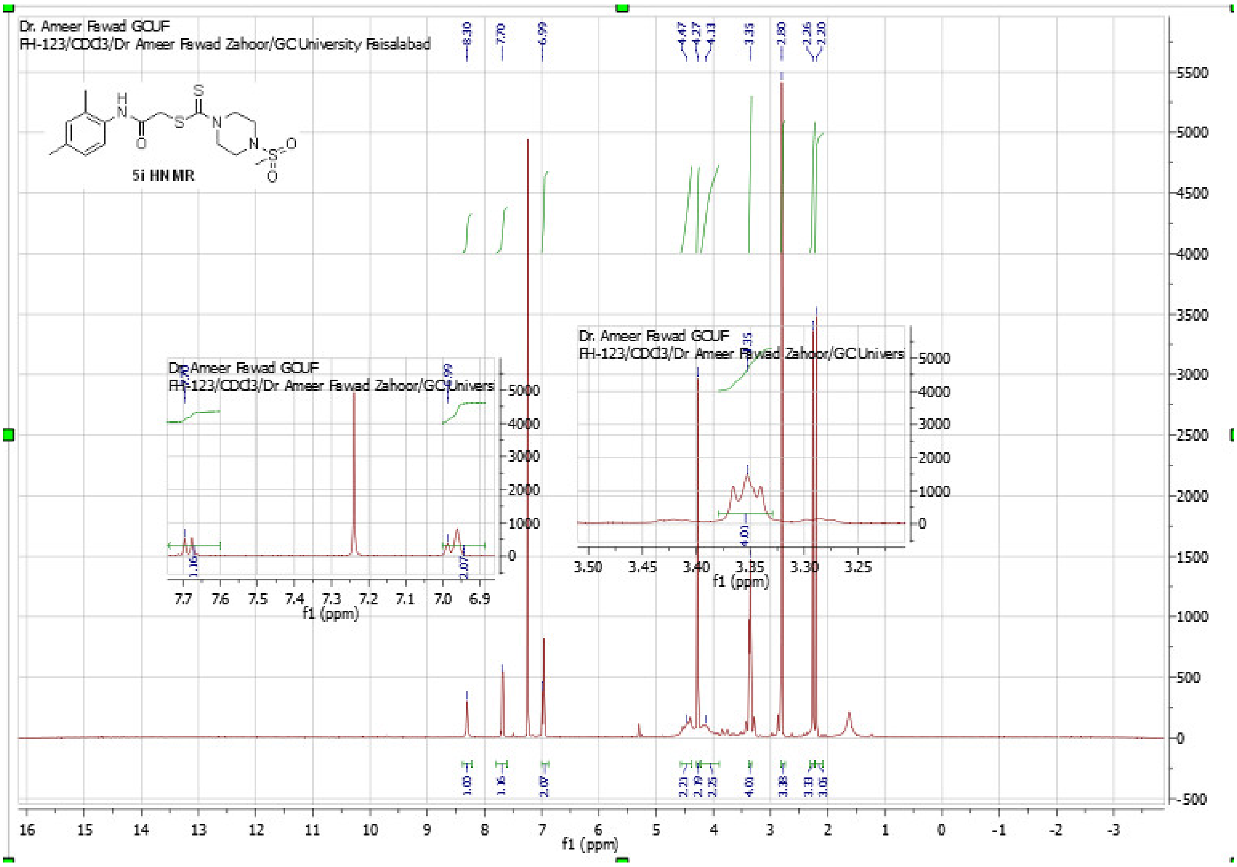

Figure S17. ^{1}H NMR of compound **5i**.

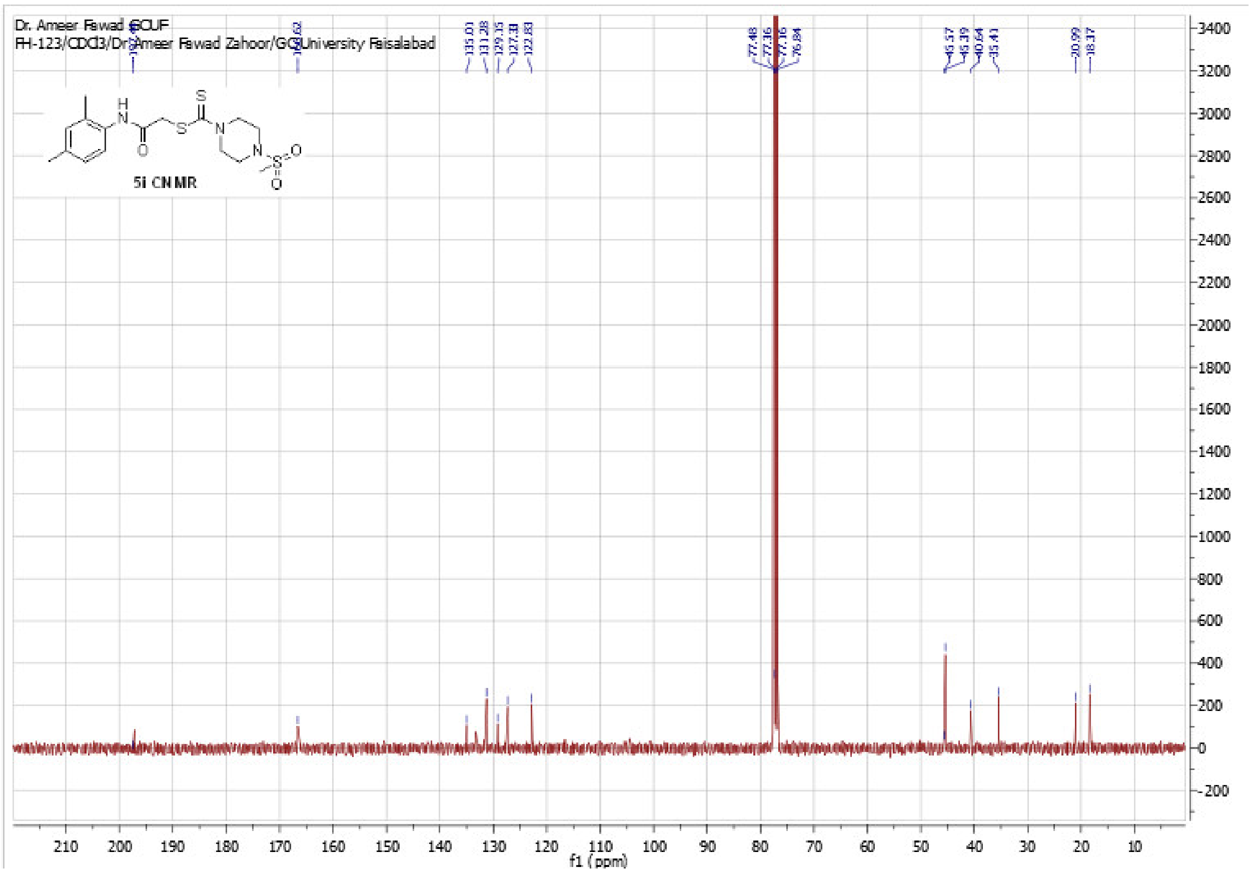

Figure S18. ^{13}C NMR of compound **5i**.

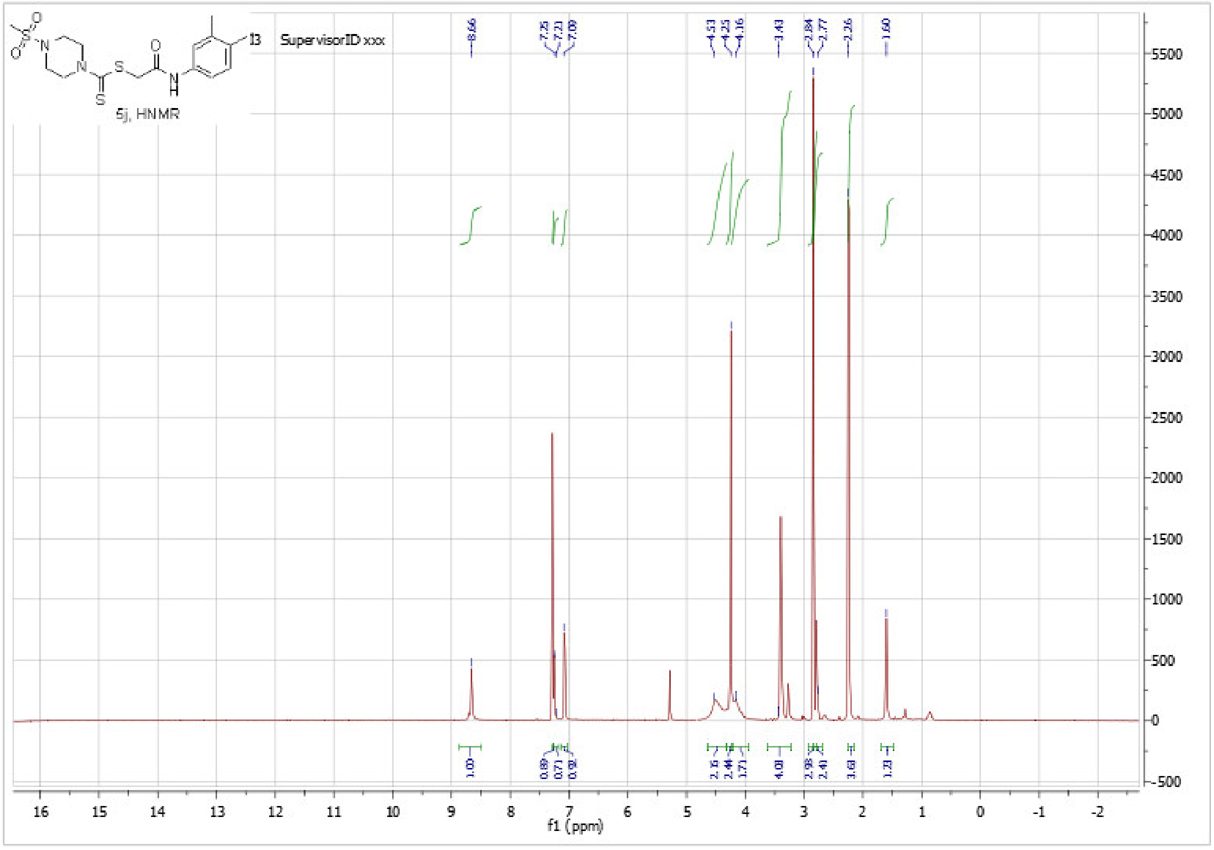

Figure S19. ^{1}H NMR of compound **5j**.

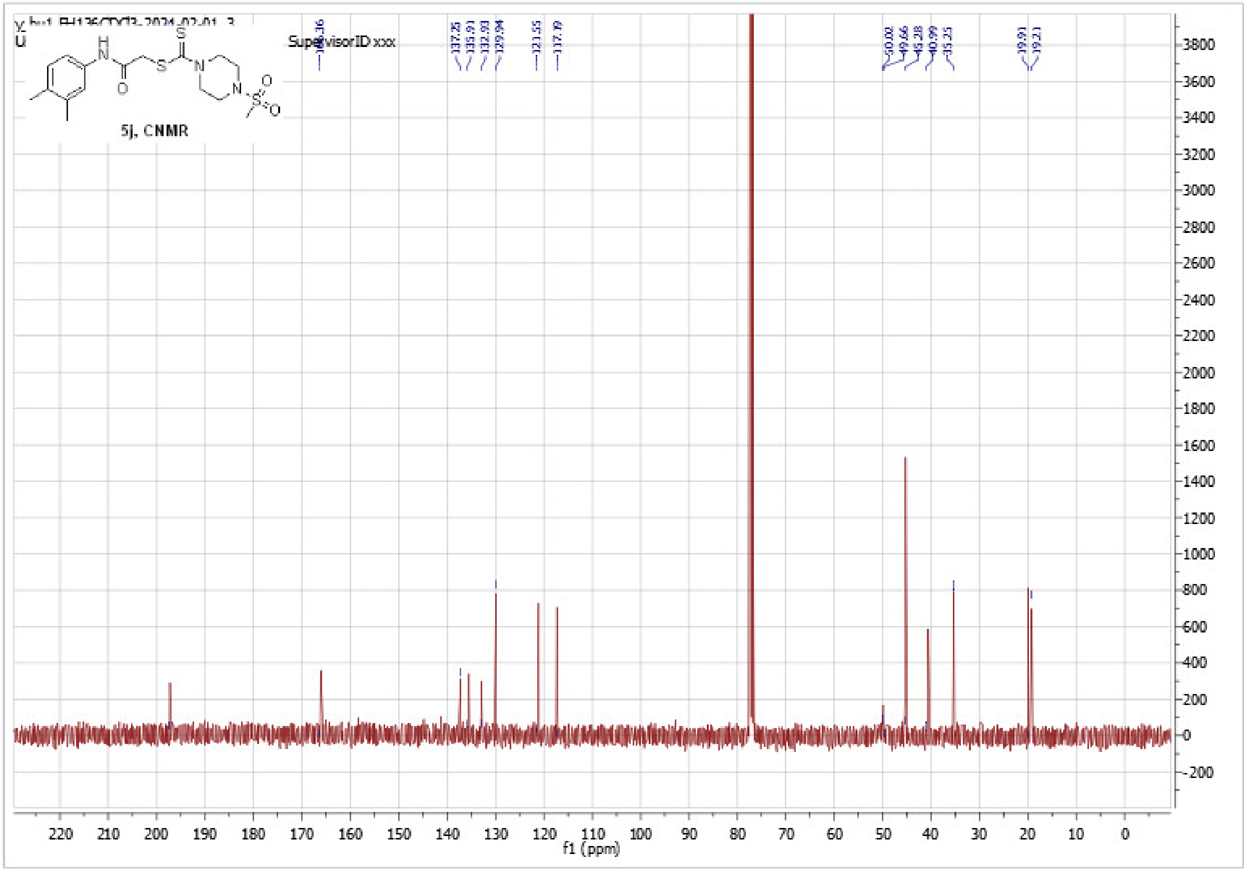

Figure S20. ^{13}C NMR of compound **5j**.
